# Supplementary figures and images for: Hybrid Machine Learning Approach to Zero-Inflated Data Improves Accuracy of Dengue Prediction
Source: PLoS Negl Trop Dis. 2024 Oct 21;18(10):e0012599. doi: 10.1371/journal.pntd.0012599 (PMC11527386; doi:10.1371/journal.pntd.0012599)

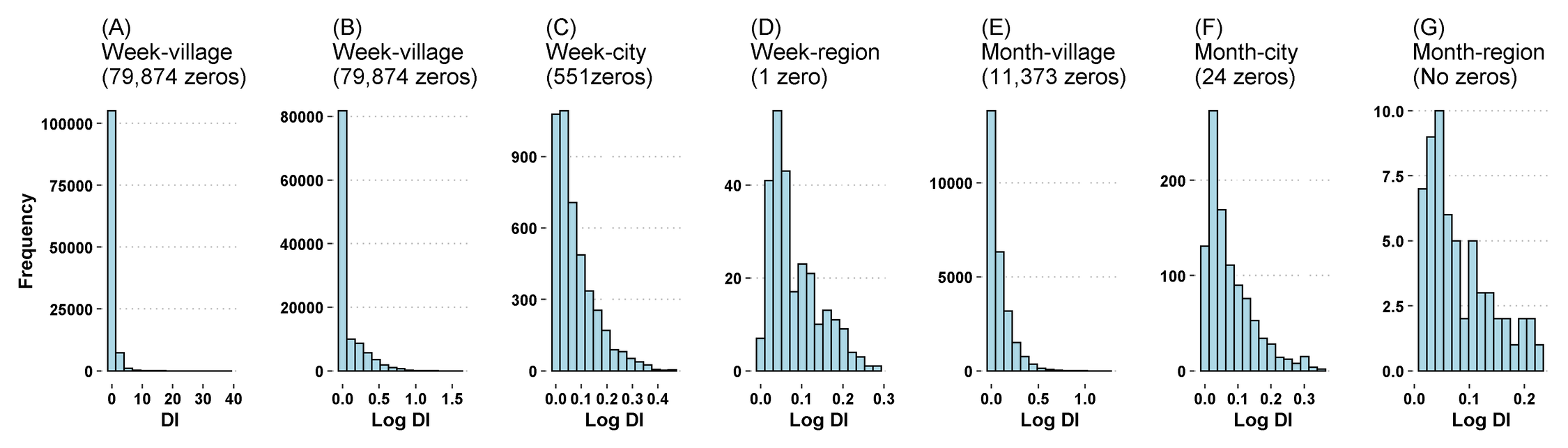

Supplement: S1 Fig — DI means Dengue Incidence. (TIF) [file pntd.0012599.s001.tif]

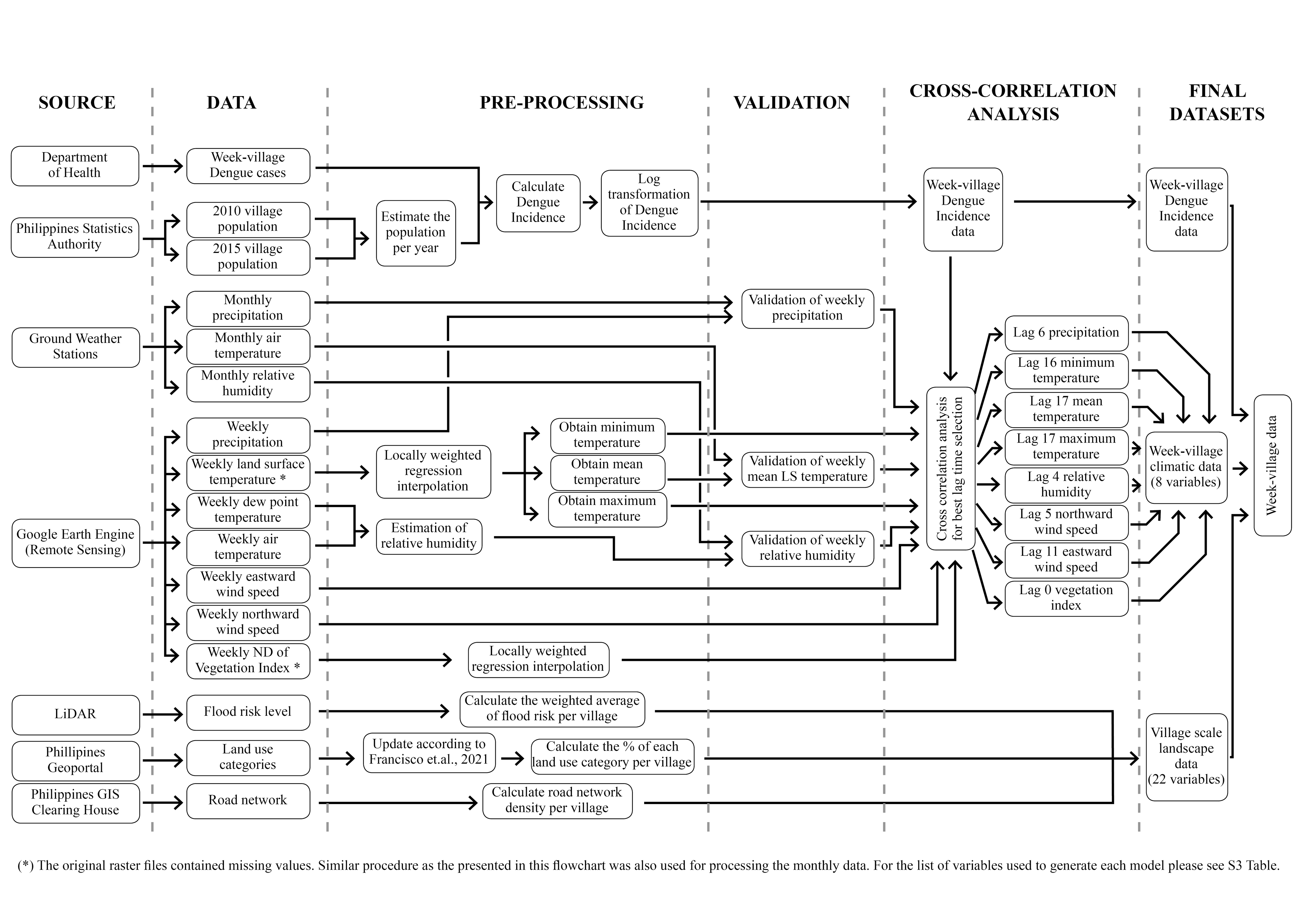

Supplement: S2 Fig — (TIF) [file pntd.0012599.s002.tif]

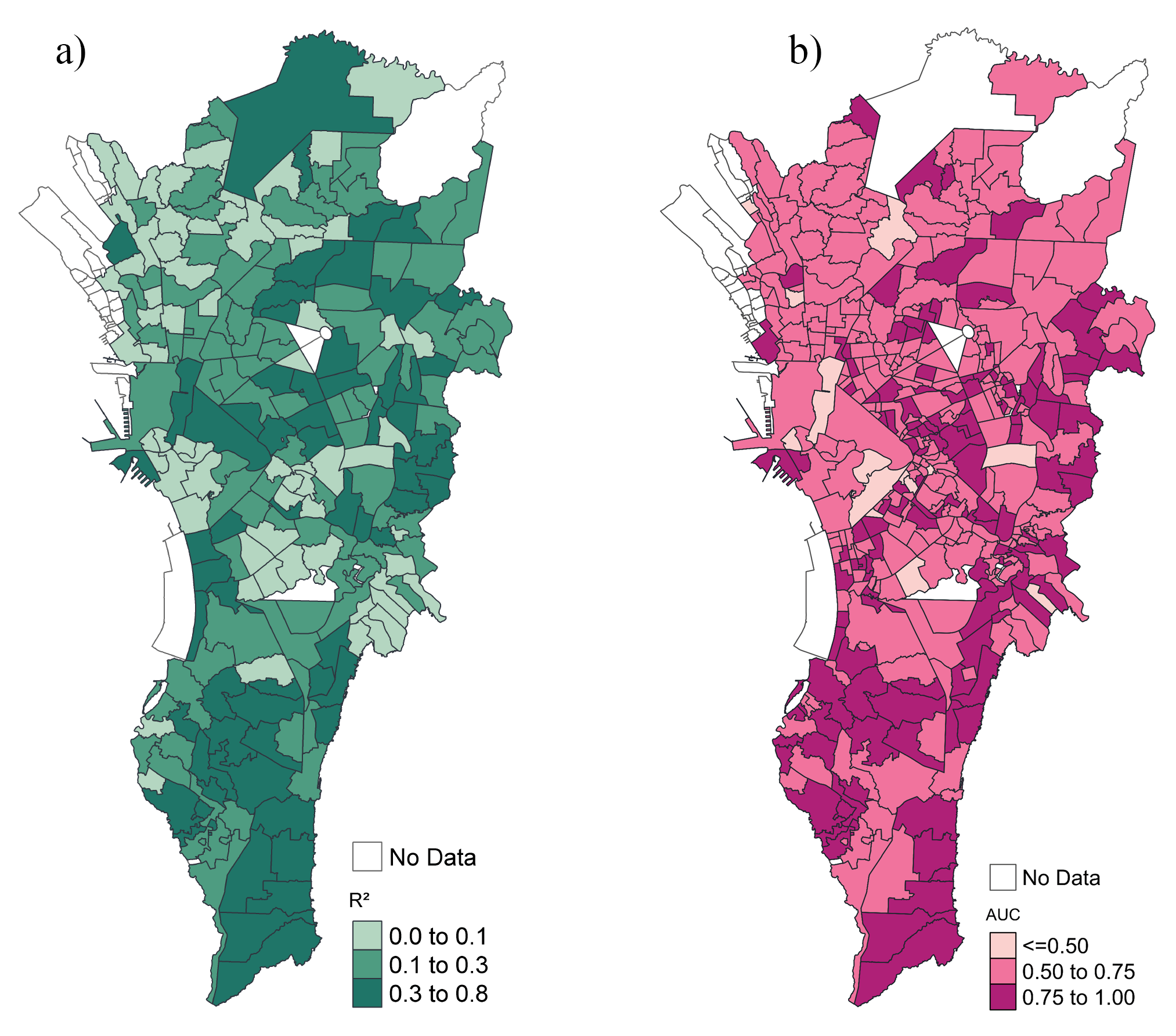

Supplement: S3 Fig — The maps baselayer are village boundaries obtained from https://data.humdata.org/dataset/cod-ab-phl. The license terms can be found here https://data.humdata.org/faqs/terms. The maps were created using the “tmap” package in R. (TIF) [file pntd.0012599.s003.tif]

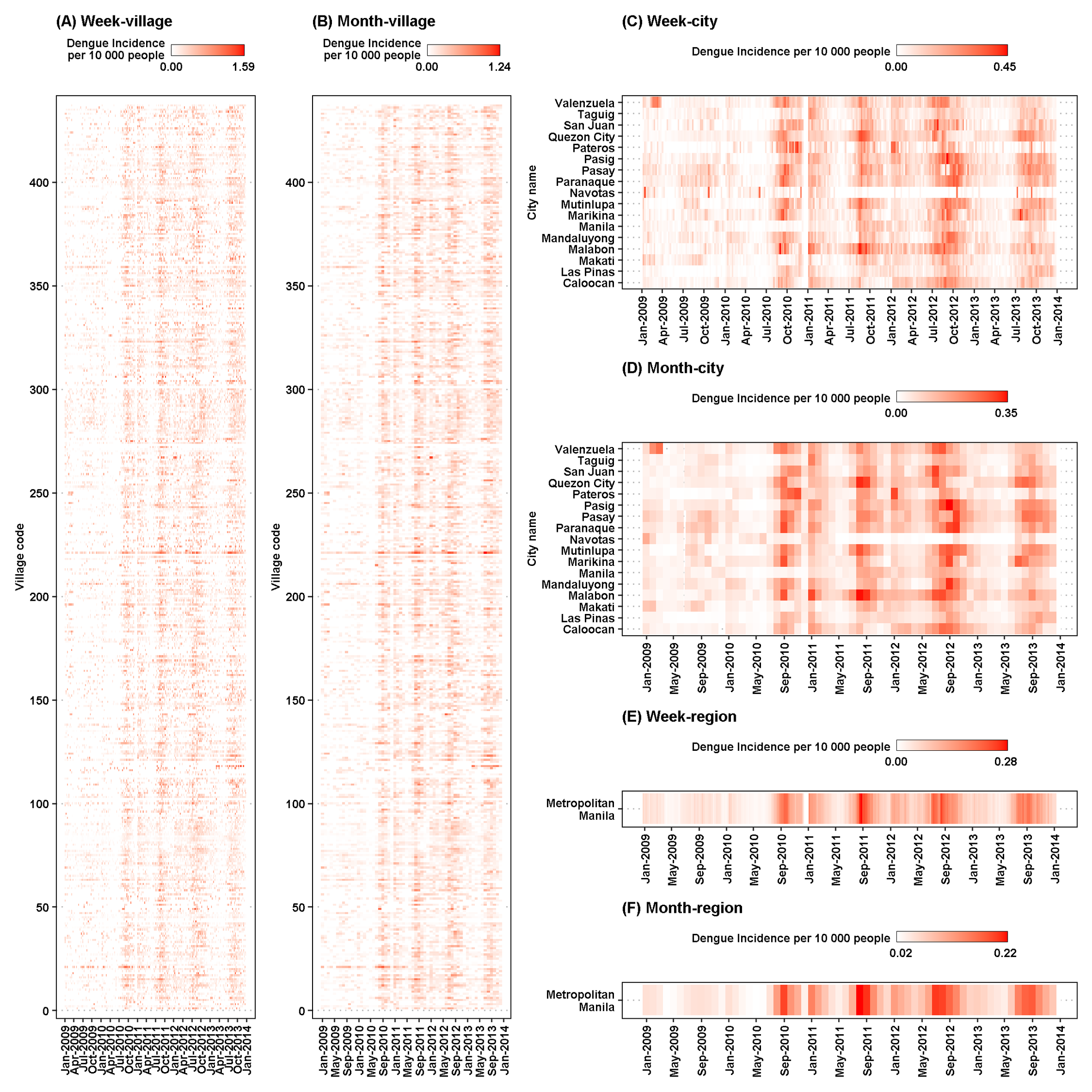

Supplement: S4 Fig — (TIF) [file pntd.0012599.s004.tif]
