# Supplementary material for: Hybrid Machine Learning Approach to Zero-Inflated Data Improves Accuracy of Dengue Prediction
Source: PLoS Negl Trop Dis. 2024 Oct 21;18(10):e0012599. doi: 10.1371/journal.pntd.0012599 (PMC11527386; doi:10.1371/journal.pntd.0012599)
Supplement: S1 Table — (DOCX) [file pntd.0012599.s005.docx]

**S3 Table.** **Characteristics of satellite data obtained from Google Earth Engine**

| Data | Product ID | Bands | Spatial resolution | Temporal resolution | Unit |
| --- | --- | --- | --- | --- | --- |
| Precipitation | NASA/GPM_L3/IMERG_V06 | precipitationCal | 11.132km | Hourly | mm/h |
| Land Surface Temperature | MODIS/006/MOD11A1 | LST_Day_1km  LST_Night_1km | 1km | Daily | K |
|  | MODIS/006/MYD11A1 | LST_Day_1km  LST_Night_1km | 1km | Daily | K |
| Dew-point temperature | ECMWF/ERA5_LAND/HOURLY | dewpoint_temperature_2m | 11.132km | Hourly | K |
| Air Temperature | ECMWF/ERA5_LAND/HOURLY | temperature_2m | 11.132km | Hourly | K |
| Wind speed | ECMWF/ERA5_LAND/HOURLY | u_component_of_wind_10m | 11.132km | Hourly | m/s |
|  | ECMWF/ERA5_LAND/HOURLY | v_component_of_wind_10m | 11.132km | Hourly | m/s |
| Vegetation | MODIS/006/MOD13Q1 | NDVI | 250m | 16-day | – |
|  | MODIS/006/MYD13Q1 | NDVI | 250m | 16-day | – |
