# Supplementary material for: Hybrid Machine Learning Approach to Zero-Inflated Data Improves Accuracy of Dengue Prediction
Source: PLoS Negl Trop Dis. 2024 Oct 21;18(10):e0012599. doi: 10.1371/journal.pntd.0012599 (PMC11527386; doi:10.1371/journal.pntd.0012599)
Supplement: S2 Table — (DOCX) [file pntd.0012599.s006.docx]

**S5 Table. Weekly and monthly cross-correlation analysis of predictors to dengue incidence**

| Variable | Week | | | Month | | |
| --- | --- | --- | --- | --- | --- | --- |
|  | Best lag | r | p-value | Best lag | r | p-value |
| Minimum temperature | 16 | 0.203 | <0.001 | 3 | 0.283 | <0.001 |
| Maximum temperature | 17 | 0.188 | <0.001 | 3 | 0.257 | <0.001 |
| Mean temperature | 17 | 0.203 | <0.001 | 3 | 0.280 | <0.001 |
| Precipitation | 6 | 0.151 | <0.001 | 1 | 0.302 | <0.001 |
| Relative humidity | 4 | 0.208 | <0.001 | 0 | 0.308 | <0.001 |
| Northward wind speed | 5 | 0.183 | <0.001 | 1 | 0.281 | <0.001 |
| Eastward wind speed | 11 | 0.143 | <0.001 | 2 | 0.229 | <0.001 |
| Vegetation index | 0 | -0.056 | <0.001 | 0 | -0.076 | <0.001 |
