# Supplementary material for: Hybrid Machine Learning Approach to Zero-Inflated Data Improves Accuracy of Dengue Prediction
Source: PLoS Negl Trop Dis. 2024 Oct 21;18(10):e0012599. doi: 10.1371/journal.pntd.0012599 (PMC11527386; doi:10.1371/journal.pntd.0012599)
Supplement: S3 Table — (DOCX) [file pntd.0012599.s007.docx]

**S6 Table. Variables used for the implementation of models**

| Model and reference results | Variables used |
| --- | --- |
| - Week-village quantitative models (Table 1) - Week-village qualitative models (Table 2) - Week-village (aggregated) quantitative models (S4) - Week-village (aggregated) qualitative models (S5) - Week-city quantitative models (Table 1) - Month-village quantitative models (Table 1) - Month-city quantitative models (Table 1) - Week-merged villages quantitative models (S4) - Week-village hybrid models (Table 4) | Minimum temperature (ºC)  Maximum temperature (ºC)  Mean temperature (ºC)  Precipitation (mm/h)  Relative humidity (%)  Northward wind speed (m/s)  Eastward wind speed (m/s)  Vegetation Index  Level of flood risk  Road network density (m/m^2^)  Percentage of agricultural (%)  Percentage of grasslands (%)  Percentage of forest lands (%)  Percentage of surface covered by water bodies (%)  Percentage of open spaces (%)  Percentage of parks and recreation areas (%)  Percentage of educational areas (%)  Percentage of health-related areas (%)  Percentage of cemetery areas (%)  Percentage of military areas (%)  Percentage of government areas (%)  Percentage of industrial areas (%)  Percentage of commercial areas (%)  Percentage of transportation areas (%)  Percentage of informal settlement areas (%)  Percentage of very low residential density areas (%)  Percentage of low residential density areas (%)  Percentage of medium residential density areas  Percentage of high residential density areas (%)  Percentage of very high residential density areas (%) |
| - Week-region quantitative models (Table 1) - Month-region quantitative models (Table 1) - Village temporal models (Fig. 1) - Aggregated villages temporal models (S6) | Minimum temperature (ºC)  Maximum temperature (ºC)  Mean temperature (ºC)  Precipitation (mm/h)  Relative humidity (%)  Northward wind speed (m/s)  Eastward wind speed (m/s)  Vegetation Index |
