# Supplementary material for: Hybrid Machine Learning Approach to Zero-Inflated Data Improves Accuracy of Dengue Prediction
Source: PLoS Negl Trop Dis. 2024 Oct 21;18(10):e0012599. doi: 10.1371/journal.pntd.0012599 (PMC11527386; doi:10.1371/journal.pntd.0012599)
Supplement: S4 Table — (DOCX) [file pntd.0012599.s008.docx]

**S9 Table. Quantitative model accuracy changes with aggregating villages.** RMSE = root mean squared error, S-Ra² = standardized adjusted R-squared, train obs. = number of observations used for training, test obs. = number of observations in model validation.

| Algorithm | Non-merged villages dataset  (train obs. = 90896, test obs. = 22724, predictors = 30) | | Merged villages dataset  (train obs. = 47008, test obs. = 11752, predictors = 30) | |
| --- | --- | --- | --- | --- |
|  | RMSE | S-R_a_² | RMSE | S-R_a_² |
| GAM | 0.165 | 0.081 | 0.112 | 0.147 |
| RF | 0.163 | 0.102 | 0.109 | 0.191 |
| CIF | 0.161 | 0.116 | 0.107 | 0.220 |
| ANN | 0.179 | 0.036 | 0.109 | 0.181 |
| SVR | 0.165 | 0.077 | 0.114 | 0.185 |
| XGB | 0.228 | 0.103 | 0.197 | 0.221 |
