# Supplementary material for: Hybrid Machine Learning Approach to Zero-Inflated Data Improves Accuracy of Dengue Prediction
Source: PLoS Negl Trop Dis. 2024 Oct 21;18(10):e0012599. doi: 10.1371/journal.pntd.0012599 (PMC11527386; doi:10.1371/journal.pntd.0012599)
Supplement: S5 Table — (DOCX) [file pntd.0012599.s009.docx]

**S10 Table. Qualitative model accuracy changes with aggregating villages.** AUC = arena under the curve, Sens. = sensitivity, Spec. = specificity, train obs. = number of observations used for training, test obs. = number of observations in model validation.

| Algorithm | Non-merged villages dataset  (train obs. = 90896, test obs. = 22724, predictors = 30) | | | Merged villages dataset  (train obs. = 47008, test obs. = 11752, predictors = 30) | | |
| --- | --- | --- | --- | --- | --- | --- |
|  | AUC | Sens. | Spec. | AUC | Sens. | Spec. |
| GAM | 0.689 | 0.591 | 0.675 | 0.725 | 0.732 | 0.605 |
| RF | 0.799 | 0.748 | 0.745 | 0.797 | 0.724 | 0.719 |
| CIF | 0.794 | 0.732 | 0.745 | 0.806 | 0.751 | 0.696 |
| ANN | 0.715 | 0.699 | 0.692 | 0.773 | 0.750 | 0.652 |
| SVM | 0.753 | 0.779 | 0.717 | 0.794 | 0.771 | 0.678 |
| XGB | 0.791 | 0.717 | 0.751 | 0.808 | 0.749 | 0.704 |
