# Supplementary material for: Hybrid Machine Learning Approach to Zero-Inflated Data Improves Accuracy of Dengue Prediction
Source: PLoS Negl Trop Dis. 2024 Oct 21;18(10):e0012599. doi: 10.1371/journal.pntd.0012599 (PMC11527386; doi:10.1371/journal.pntd.0012599)
Supplement: S1 Text — (DOCX) [file pntd.0012599.s010.docx]

**S1 Text. Model approach and implementation**

**Generalized Additive Model (GAM)**

GAM is an improvement of linear models which replaces the linear form with a sum of smooth functions [1]. In GAM, the impact of the predictive variables can be captured through smooth functions which depend on the underlying patterns in the data that can be nonlinear. The incorporation of smooth functions to at least one linear predictor (or all) will allow to effectively capture nonlinear relationships between the covariates and the target variable. Another advantage of GAMs is easy interpretability which can be biased in linear models and the “black box” machine learning algorithms. Because the performance of a model can be sensitive to tuning parameters, we performed a grid search by looping a model implementation over several parameters combinations in all algorithms. The loop trains several models by gradually supplying and increasing the values of the model parameters. These parameters may change in both type and number depending on the algorithm. In GAM the models were tuned over a vector of smoothing parameters. In all cases, the other parameters were set as the default as per package configuration. GAM was implemented in R package “mgcv” version 1.8-35 [2].

**Random Forests (RF)**

RF is a bootstrap aggregation (bagging) ensemble method that generates a large number of independent bootstrapped trees from random small subsets of the dataset [3]. The prediction with the most votes from each individual tree becomes the model’s prediction. Since each tree is generated from a different bootstrapped sample, it is expected that the trees protect each other from their individual errors contributing for a high model accuracy. A total of 4 parameters were tuned namely number of variables (mtry), number of trees (ntree), percentage of bootstrap sample and node size. RF was implemented thru the R package “ranger” version 0.13.1 [4].

**Conditional Inference Forests (CIF)**

Unlike the original RF, CIF employ conditional inference tree (Ctree) as the base learner. When fitting a Ctree, the conditional distribution of the statistics that measure the associations between the response and the predictor variables is derived. Multiple testing procedures (adjusted P-values) are applied to determine whether there exist statistically significant associations between any of the predictors and the response [5]. The optimal split-variable in the beginning partitioning is obtained by testing the association of all the covariates to the outcome using an appropriate linear rank test [6]. The forest of conditional inference trees results into a conditional inference (CIF) model. Following is the summary of steps to that outlines the general algorithm for building a conditional inference tree as presented by [6]: (1) For case weights, test the global null hypothesis of independence between any of the covariates and the response. Stop if this hypothesis cannot be rejected. Otherwise select the covariate with strongest association to the response. (2) Choose a subset in order to split that significant covariate into two disjoint subsets. (3) Recursively repeat steps 1 and 2 with modified case weights on both subsets respectively. For CIF only number of variables (mtry) and number of trees (ntree) were tuned in “mlr” R package version 2.19.0. [7].

**Extreme Gradient Boosting (XGB)**

XGB is one of the implementations of gradient boosting machines framework which is known as one of the best performing tree-based algorithms utilized for supervised learning [8]. It is widely used for both regression and classification problems. XGB has notable advantages as is one of the preferred by data scientists because its high execution speed out of core computation [9]. Unlike RF which constructs independent trees, in XGB, ensemble trees are constructed with additive training functions to predict the output. As a result, the trees are successively optimized in order to improve the accuracy. Tuning was performed over the max number of iterations (nround), maximum depth of the tree (max.depth) and step size of each boosting step (eta) using the “xgboost” package version 1.5.0.2 [9] in R.

**Artificial Neural Networks (ANN)**

ANN are models that involves computations and mathematics, which simulate the human–brain processes. Like the structure of the human brain, the ANN models consist of neurons in a complex and nonlinear form. The neurons are connected to each other by weighted links. ANN model comprise at least three layers: input, hidden, and output [10]. Computation of ANN begins with introducing an array of numbers to the input layer of the processing nodes. These signals then move along connections to each of the nodes in the adjacent layer and can be inhibited or amplified through connection-specific weights. All of the weight-adjusted input values to a processing element are then aggregated using a vector to scalar function to produce a single input value to the neuron. Once the input value is calculated, the processing element then uses a transfer function to produce its output. The transfer function on its turn transforms the input value using a sigmoidal, hyperbolic-tangent or other nonlinear activation function [11]. The process is repeated between layers until a final output value or vector of values is produced [12]. For ANN number of units in the hidden layer (size), maximum number of iterations (maxit) and the maximum allowable number of weights (MaxNWts) were tuned for the model fit using the “nnet” R package version 7.3-16 [11].

**Support Vector Machines (SVM)**

SVM extend to non-linear classification by projecting the original input space into a high-dimensional space where the classes of a variable can be separated by hyperplane. Hyperplanes are decision boundaries that help classify the data points by maximizing the distance between those classes [13]. Data points falling on either side of the hyperplane can be attributed to different classes. The Support Vector Regression (SVR) is based on SVM concept, intended for regression analysis. In SVR, the model is fit by considering only the points that falls in the decision boundary whose have least error rate in order to improve accuracy [14]. Models were tuned over the gamma, epsilon and the parameter needed for the kernel (coef0) in R package “e1071” version 1.7-9 [15]. The sample code for the implementation of each algorithm is added in S1 Code.

# References

| [1] | T. Hastie and R. Tibshirani, "Generalized Additive Models," *Statistical Science,* vol. 1, no. 3, pp. 297-318, 1986. |
| --- | --- |
| [2] | S. Wood, "Package ‘mgcv’," 2021. [Online]. Available: https://cran.r-project.org/web/packages/mgcv/mgcv.pdf. [Accessed 6 12 2021]. |
| [3] | L. Breiman, "Random Forests," *Machine Learning,* pp. 5-32, 2001. |
| [4] | M. N. Wright, S. Wager and P. Probst, "A Fast Implementation of Random Forests," 10 1 2020. [Online]. Available: https://cran.r-project.org/web/packages/ranger/ranger.pdf. |
| [5] | R. Xia, "Comparison of Random Forests and Cforest: Variable Importance Measures and Prediction Accuracies," All Graduate Plan B and other Reports, 2009. |
| [6] | T. Hothorn, K. Hornik and A. Zeileis, "Unbiased Recursive Partitioning: A Conditional Inference Framework," *Journal of Computational and Graphical Statistics,* vol. 15, no. 3, pp. 651-674, 2006. |
| [7] | B. Bischl, M. Lang, L. Kotthoff, P. Schratz, J. Schiffner, J. Richter, Z. Jones and G. Casalicchio, "Package ‘mlr’," 2021. [Online]. Available: https://cran.r-project.org/web/packages/mlr/mlr.pdf. [Accessed 10 11 2021]. |
| [8] | A. I. A. Osman, A. N. Ahmed, M. F. Chow, Y. F. Huang and A. El-Shafie, "Extreme gradient boosting (Xgboost) model to predict the groundwater levels in Selangor Malaysia," *Ain Shams Engineering Journal,* vol. 12, no. 2, pp. 1545-1556, 2021. |
| [9] | T. Chen and C. Guestrin, "XGBoost: A Scalable Tree Boosting System," *Association for Computing Machinery,* pp. 785-794, 2016. |
| [10] | K. Gurney, An introduction to neural networks, London and New York: ROUTLEDGE, 2003. |
| [11] | S. Walczak and N. Cerpa, Artificial Neural Networks, Third Edition ed., R. A. Meyers, Ed., Academic Press, 2003, pp. 631-645. |
| [12] | M. K. Tiwari, R. C. Deo and J. F. Adamowski, "Short-term flood forecasting using artificial neural networks, extreme learning machines, and M5 model tree," in *Advances in Streamflow Forecasting*, D. M. Priyanka Sharma, Ed., Elsevier, 2021, pp. 263-279. |
| [13] | M. Awad and R. Khanna, "Support Vector Machines for Classification. In: Efficient Learning Machines," Berkeley, CA, Apress, 2015. |
| [14] | D. Basak, S. Pal and D. C. Patranabis, "Support Vector Regression," *Neural Information Processing,* vol. 11, no. 10, pp. 203-224, 2007. |
| [15] | D. Meyer, E. Dimitriadou, K. Hornik, A. Weingessel, F. Leisch, C.-C. Chang and C.-C. Lin, "Package ‘e1071’," 2021. [Online]. Available: https://cran.r-project.org/web/packages/e1071/e1071.pdf. [Accessed 8 8 2021]. |
