# Supplementary material for: Hybrid Machine Learning Approach to Zero-Inflated Data Improves Accuracy of Dengue Prediction
Source: PLoS Negl Trop Dis. 2024 Oct 21;18(10):e0012599. doi: 10.1371/journal.pntd.0012599 (PMC11527386; doi:10.1371/journal.pntd.0012599)
Supplement: S1 Code — (DOCX) [file pntd.0012599.s011.docx]

**S8 Code. Code for the implementation of the hybrid model**

**SAMPLE CODE**

####DATA PREPARATION####

#Set the Working directory

setwd**(**"~"**)**

#import the data with continuous outcome

Initial.Data **<-** read.csv**(**"Continuous_Data.csv"**)**

#Transform the continous outcome to a binary outcome

Bin_Target **<-** ifelse**(**Initial.Data**$**Cont_Target **==** 0,0,1**)**

Bin_Target

Bin.Data **<-** cbind**(**Bin_Target, Initial.Data**[**2**:**ncol**(**Initial.Data**)])**

####STEP 1####

#Implement the first step: Fit the model to predict 0-1 (Qualitative model)

Bin.Train **<-** Bin.Data**[**1**:**400,**]** #Set according to data

Bin.Test **<-** Bin.Data**[**401**:**nrow**(**Bin.Data**)**,**]** #Set according to data

#Fit the qualitative model. Here we give example of randomForest in ranger package

#For brevity in this example we do not include model tunning

# The model can be tunned for best hyperparameters according to the algorithm in use based on the R package documentation

library**(**ranger**)**

Step1.Model **<-** ranger**(**Bin_Target **~**.,

classification **=** T,

num.trees **=** 500,

mtry **=** 3,

data **=** Bin.Train**)**

Step1.Predictions **<-** predict**(**Step1.Model, Bin.Test**)**

Step1.Predicted.Data **<-** data.frame**(**Step1.Predictions**$**predictions, Bin.Test**$**Bin_Target**)**

#Calculate the AUC

library**(**pROC**)**

Step1.ROC **<-** roc**(**as.vector**(**Step1.Predictions**$**predictions**)**, Bin.Test**$**Bin_Target**)**

Step1.AUC **<-** as.numeric**(**auc**(**Step1.ROC**))** # AUC Value between 0 to 1

####STEP 2####

#Implement the second step; Fit the model to predict the continuous values (Quantitative model)

Cont.Train_ **<-** Initial.Data**[**1**:**400,**]** #Split Set according to data

Cont.Test_ **<-** Initial.Data**[**401**:**nrow**(**Initial.Data**)**,**]** #Split Set according to data

library**(**dplyr**)**

Cont.Train **<-** Cont.Train_%>% filter**(**Cont_Target **>** 0**)** #Filter only the non-zero values

#Fit the quantitative model. Here we give example of randomForest in ranger package

#For brevity in this example we do not include model tunning

# The model can be tunned for best hyperparameters according to the algorithm in use based on the R package documentation

Step2.Model **<-** ranger**(**Cont_Target**~**.,

num.trees **=** 300,

mtry **=** 3,

data **=** Cont.Train**)** #make sure not to use Cont.Train_ as it still has zero values

#Get those observations from the test set that were predicted as 1 and join with the original continuous values

Cont.Test **<-** cbind**(**Cont.Test_,

data.frame**(**Step1.Predictions **=** Step1.Predicted.Data**$**Step1.Predictions.predictions**))**

Cont.Test **<-** Cont.Test %>% filter**(**Step1.Predictions **==** 1**)**

Cont.Test **<-** Cont.Test**[**, **-**ncol**(**Cont.Test**)]**

Step2.Predictions **<-** predict**(**Step2.Model, Cont.Test**)**

Step2.Predicted.Data **<-** data.frame**(**Step2.Predictions**$**predictions, Cont.Test**$**Cont_Target**)**

#Calculate the R^2

Step2.R2 **<-** cor**(**Step2.Predicted.Data**$**Step2.Predictions.predictions, Step2.Predicted.Data**$**Cont.Test.Cont_Target**)^**2

####HAI####

#Calculate the Hybrid Accuracy Index (HAI)

Number.zeros **<-** Bin.Data %>% count**(**Bin.Data**$**Bin_Target **==** 0**)**

Percentage.zeros **<-** Number.zeros**[**2,2**]/**nrow**(**Bin.Data**)**

Number.non_zeros **<-** Initial.Data %>% count**(**Initial.Data**$**Cont_Target **>** 0**)**

Percentage.non_zeros **<-** Number.non_zeros**[**2,2**]/**nrow**(**Initial.Data**)**

HAI **<-** **(**Step1.AUC*****Percentage.zeros**)** **+** **(**Step2.R2*****Percentage.non_zeros**)**

HAI

**ACTUAL CODE USED**

####HAI####

#Calculate the Hybrid Accuracy Index (HAI)

##### PREPARATION #####

setwd**(**""**)**

getwd**()**

#load the data

WV_row **<-** read.csv**(**"final_pv_vil_week.csv"**)**

head**(**WV_row**)**

dim**(**WV_row**)**

#Convert continuous dengue incidence to a binary variable

biden **<-** data.frame**(**biden **=** ifelse**(**WV_row**$**logden **>** 0,1,0**))**

#Bind the binary variable with environmental predictors

bin_WV **<-** cbind**(**biden, WV_row**[**7**:**ncol**(**WV_row**)])**

bin_WV**$**biden **<-** as.factor**(**bin_WV**$**biden**)**

head**(**bin_WV**)**

dim**(**bin_WV**)**

#Split data into train and test

bin_train **<-** bin_WV**[**1**:**90896,**]** #This corresponds to 2009 - 2012 data

bin_test **<-** bin_WV**[**90897**:**nrow**(**bin_WV**)**,**]** #This corresponds to 2013 data

head**(**bin_train**)**

head**(**bin_test**)**

dim**(**bin_train**)**

dim**(**bin_test**)**

#Load all necessary Packages

library**(**e1071**)** #For ANN

library**(**nnet**)** #For ANN

library**(**mgcv**)** # For GAM

library**(**mlr**)** #For CIF

library**(**ranger**)** #For RF

library**(**xgboost**)** #For XGB

library**(**caret**)** #For tunning

library**(**dplyr**)** #For data manipulation

library**(**party**)** #For CIF

library**(**Metrics**)**#for RMSE

library**(**pROC**)** #For calculating ROC/AUC

#### PART I - QUALITATIVE MODELS ######

###### Part I - GAM ######

srt.gam **<-** Sys.time**()**

#fit a base model

Ql_GAM **<-** gam**(**biden **~** **(**Edu**)+(**Ind**)+(**Hig**)+**s**(**tmin_L16**)+**

**(**tmean_L17**)+(**uw_L5**)+(**Hea**)+(**Com**)+**

**(**Vhi**)+(**tmax_L17**)+(**ndvi_L0**)+(**vw_L11**)+**

**(**Cem**)+(**Tra**)+(**RND**)+(**l_sgpm_L6**)+(**rh_L4**)+**

**(**Mil**)+(**Inf.**)+(**FloodRisk**)+(**Gov**)+(**Vlo**)+**

**(**Low**)+(**Agr**)+(**Gra**)+(**For**)+(**Wat**)+(**Ope**)+(**Par**)**,

data **=** bin_train, family **=** "binomial"**)**

summary**(**Ql_GAM**)**

#prepare for model tunning

sp **<-** Ql_GAM**$**sp

tuning.scale**<-**c**(**1e-5,1e-4,1e-3,1e-2,1e-1,1e0,1e1,1e2,1e3,1e4,1e5**)**

scale.exponent**<-**log10**(**tuning.scale**)**

n.tuning**<-**length**(**tuning.scale**)**

edf**<-**rep**(NA**,n.tuning**)**

min2ll**<-**rep**(NA**,n.tuning**)**

aic**<-**rep**(NA**,n.tuning**)**

bic**<-**rep**(NA**,n.tuning**)**

#tune the model

**for** **(**i **in** 1**:**n.tuning**)** **{**

Ql_GAM **<-**gam**(**biden **~** **(**Edu**)+(**Ind**)+(**Hig**)+**s**(**tmin_L16**)+**

**(**tmean_L17**)+(**uw_L5**)+(**Hea**)+(**Com**)+**

**(**Vhi**)+(**tmax_L17**)+(**ndvi_L0**)+(**vw_L11**)+**

**(**Cem**)+(**Tra**)+(**RND**)+(**l_sgpm_L6**)+(**rh_L4**)+**

**(**Mil**)+(**Inf.**)+(**FloodRisk**)+(**Gov**)+(**Vlo**)+**

**(**Low**)+(**Agr**)+(**Gra**)+(**For**)+(**Wat**)+(**Ope**)+(**Par**)**,

sp**=**tuning.scale**[**i**]***sp,

data **=** bin_train,

family **=** binomial**(**link**=logit))**

min2ll**[**i**]<--**2*****logLik**(**Ql_GAM**)**

edf**[**i**]<-**sum**(**Ql_GAM**$**edf**)+**1

aic**[**i**]<-**AIC**(**Ql_GAM**)**

bic**[**i**]<-**BIC**(**Ql_GAM**)**

print**(**paste**(**"Model fitted. LogLik:", min2ll**[**i**]**,

"EDF:", edf**[**i**]**, "AIC:", aic**[**i**]**, "BIC:", bic**[**i**]))**

**}**

#visualizations

par**(**mfrow**=**c**(**2,2**)**, mar**=**c**(**4, 4, 2, 1**))** # Adjust the 'mar' parameter as needed

plot**(**scale.exponent, min2ll, type**=**"b", main**=**"2 log likelihood"**)**

plot**(**scale.exponent, edf, ylim**=**c**(**0,70**)**, type**=**"b",

main**=**"effective number of parameters"**)**

plot**(**scale.exponent, aic, type**=**"b", main**=**"AIC"**)**

plot**(**scale.exponent, bic, type**=**"b", main**=**"BIC"**)**

min.bic**<-**1e100

opt.tuning.scale**<-NULL**

**for** **(**i **in** 1**:**n.tuning**)** **{**

**if** **(**bic**[**i**]<**min.bic**)** **{**

min.bic**<-**bic**[**i**]**

opt.tuning.scale**<-**tuning.scale**[**i**]**

**}**

print**(**paste**(**"Iteration:", i, "BIC:", bic**[**i**]**,

"Min BIC:", min.bic, "Optimal Tuning Scale:", opt.tuning.scale**))**

**}**

opt.sp**<-**opt.tuning.scale*****sp

#fit the model with best hyperparameters

Ql_GAM **<-**gam**(**biden **~** **(**Edu**)+(**Ind**)+(**Hig**)+**s**(**tmin_L16**)+**

**(**tmean_L17**)+(**uw_L5**)+(**Hea**)+(**Com**)+**

**(**Vhi**)+(**tmax_L17**)+(**ndvi_L0**)+(**vw_L11**)+**

**(**Cem**)+(**Tra**)+(**RND**)+(**l_sgpm_L6**)+(**rh_L4**)+**

**(**Mil**)+(**Inf.**)+(**FloodRisk**)+(**Gov**)+(**Vlo**)+**

**(**Low**)+(**Agr**)+(**Gra**)+(**For**)+(**Wat**)+(**Ope**)+(**Par**)**,

sp**=**opt.sp,

data **=** bin_train,

family **=** "binomial"**)**

summary**(**Ql_GAM**)**

#predictions

fits **=** data.frame**(**fit **=** predict**(**Ql_GAM, newdata **=** bin_test,

type **=** "response", se **=** F**))**

Ql_gam_pred **<-** as.data.frame**(**fits**)**

Ql_gam_pred **<-** data.frame**(**fit **=** Ql_gam_pred**$**fit,

biden **=** bin_test**$**biden**)**

head**(**Ql_gam_pred**)**

Ql_gam_pred**$**fit **<-** ifelse**(**Ql_gam_pred**$**fit**>=**0.5,1,0**)**

#save the predictions

write.table**(**Ql_gam_pred, "Ql_gam_pred.csv", col.names **=** T,

row.names **=** F, sep **=** ","**)**

#calculate AUC

roc_gam **<-** pROC**::**roc**(**as.vector**(**Ql_gam_pred**$**biden**)**, as.vector**(**Ql_gam_pred**$**fit**))**

auc_gam **=** cat**(**"AUC: ", pROC**::**auc**(**roc_gam**))**# AUC Value between 0 to 1

auc_gam **=** as.numeric**(**pROC**::**auc**(**roc_gam**))**

auc_gam

end.gam **<-** Sys.time**()**

time.gam **<-** end.gam **-** srt.gam

###### Part I - ANN ######

srt.ann **<-** Sys.time**()**

#tune the model

Ql_ANN **<-** tune.nnet**(**biden **~** .,

data**=**bin_train,

size**=**1**:**10, linout**=**F, skip**=**T,

MaxNWts**=**500, trace**=**T,

maxit**=**1000**)**

#check the outputs for selecting best hyperparameters

Ql_ANN

#Fit the model with best hyperparameters

Ql_ANN **<-** nnet**(**biden **~** .,

data**=**bin_train,

size**=**10, linout**=**F, skip**=**T,

MaxNWts**=**500, trace**=**T,

maxit**=**1000**)**

#predictions

predict_nnet **=** predict**(**Ql_ANN, newdata**=**bin_test**)**

Ql_ann_pred **=** data.frame**(**predict_nnet, bin_test**$**biden**)**

#save predictions

write.table**(**Ql_ann_pred, "Ql_ann_pred.csv",

col.names **=** T, row.names **=** F, sep **=** ","**)**

#calculate AUC

roc_ann **<-** pROC**::**roc**(**as.vector**(**bin_test**$**biden**)**, Ql_ann_pred**$**predict_nnet**)**

auc_ann **<-** cat**(**"AUC: ", pROC**::**auc**(**roc_ann**))** # AUC Value between 0 to 1

auc_ann **<-** as.numeric**(**pROC**::**auc**(**roc_ann**))**

auc_ann

end.ann **<-** Sys.time**()**

time.ann **<-** end.ann **-** srt.ann

###### Part I - SVM ######

srt.svm **<-** Sys.time**()**

#model tunning

Ql_SVM_ **<-** tune.svm**(**biden**~**.

, data **=** bin_train, kernel **=** "radial",

degree**=**seq**(**2.0,3.5, by **=** 0.5**)**,

coef0**=**c**(**0.001**)**, verbose **=** T, track **=** T, trace **=** T**)**

#select the best model

Ql_SVM **<-** Ql_SVM_**$**best.model

#predictions

predictedY **<-** predict**(**Ql_SVM, bin_test**)**

Ql_svm_pred **<-** data.frame**(**predictedY, bin_test**$**biden**)**

#save predictions

write.table**(**Ql_svm_pred, "Ql_svm_pred.csv",

sep **=** ",",row.names **=** F, col.names **=** T**)**

str**(**Ql_svm_pred**)**

#calculate AUC

roc_svm **<-** pROC**::**roc**(**as.vector**(**bin_test**$**biden**)**,

as.numeric**(**Ql_svm_pred**$**predictedY**))**

auc_svm **<-** cat**(**"AUC: ", pROC**::**auc**(**roc_svm**))** # AUC Value between 0 to 1

auc_svm **<-** as.numeric**(**pROC**::**auc**(**roc_svm**))**

auc_svm

end.svm **<-** Sys.time**()**

time.svm **<-** end.svm **-** srt.svm

###### Part I - Random Forest ###############

srt.rf **<-** Sys.time**()**

#set the formula

formula **<-** biden **~**Agr**+**Gra**+**For**+**Wat**+**Ope**+**Par**+**Edu**+**Hea**+**Cem**+**Mil**+**Gov**+**

Ind**+**Com**+**Tra**+**Inf.**+**Vlo**+**Low**+**Med**+**Hig**+**Vhi**+**RND**+**FloodRisk**+**tmin_L16**+**

tmax_L17**+**tmean_L17**+**ndvi_L0**+**l_sgpm_L6**+**uw_L5**+**vw_L11**+**rh_L4

#prepare the grid for model tunning

grid.rf **<-** expand.grid**(**

mtry **=** seq**(**1, 3, by **=** 1**)**,

ntree **=** seq**(**100,1000, by**=**100**)**,

node_size **=** seq**(**2, 10, by **=** 2**)**,

sampe_size **=** c**(**0.80,0.9,1.0**)**,

OOB_RMSE **=** 0**)**

# total number of combinations

nrow**(**grid.rf**)**

# train model

**for(**i **in** 1**:**nrow**(**grid.rf**))** **{**

mod **<-** ranger**(**

formula **=** formula ,

data **=** bin_train,

num.trees **=** grid.rf**$**ntree**[**i**]**,

mtry **=** grid.rf**$**mtry**[**i**]**,

min.node.size **=** grid.rf**$**node_size**[**i**]**,

sample.fraction **=** grid.rf**$**sampe_size**[**i**]**

**)**

# add OOB error to grid

grid.rf**$**OOB_RMSE**[**i**]** **<-**sqrt**(**mod**$**prediction.error**)}**

#visualize the outputs and select best hyperparameters

grid.rf %>%

dplyr**::**arrange**(**OOB_RMSE**)** %>%

head**(**10**)**

#fist the model with best hyperparameters

Ql_RF **<-** ranger**(**formula,num.trees **=** 900, mtry **=**2,

min.node.size **=** 1, sample.fraction **=** 0.9,

data **=** bin_train **)**

#predictions

rang_pred **<-** predict**(**Ql_RF, bin_test**)**

head**(**rang_pred**)**

Ql_rf_pred **<-** data.frame**(**predictions **=** rang_pred**$**predictions,

test_biden **=** bin_test**$**biden**)**

#save predictions

write.table**(**Ql_rf_pred, "Ql_rf_pred.csv",

sep **=** ",", col.names **=** T, row.names **=** F**)**

#calculate AUC

roc_rf **<-** pROC**::**roc**(**as.vector**(**as.numeric**(**Ql_rf_pred**$**predictions**))**,

as.numeric**(**Ql_rf_pred**$**test_biden**))**

auc_rf **<-** cat**(**"AUC: ", pROC**::**auc**(**roc_rf**))** # AUC Value between 0 to 1

auc_rf **<-** as.numeric**(**pROC**::**auc**(**roc_rf**))**

auc_rf

end.rf **<-** Sys.time**()**

time.rf **<-** end.rf **-** srt.rf

###### Part I - XGBoost ######

srt.gbm **<-** Sys.time**()**

#prepare the data

bin_train **<-** xgb.DMatrix**(**

data **=** data.matrix**(**subset**(**bin_train, select **=** **-**biden**))**,

label **=** data.matrix**(**bin_train**$**biden**))**

#Fit a base model

xgb **<-** xgb.cv**(**data **=** bin_train,

eta **=** 0.0001,

max_depth **=** 50,

nround **=** 10000,

subsample **=** 0.5,

colsample_bytree **=** 0.5,

objective **=** "binary:logistic",

nthread **=** 8,

nfold **=** 5,

early_stopping_rounds **=** 50,

eval_metric **=** **'logloss')**

#select the best model

best.gbm **<-** xgb**$**best_iteration

#fit the model with best hyperparameters

Ql_XGB **<-** xgboost**(**data **=** bin_train,

eta **=** 0.0001,

max_depth **=** 50,

nround **=** best.gbm,

subsample **=** 0.5,

colsample_bytree **=** 0.5,

objective **=** "binary:logistic",

nthread **=** 8,

eval_metric **=** **'logloss')**

#predictions

preds **<-** predict**(**Ql_XGB, data.matrix**(**subset**(**bin_test, select **=** **-**biden**)))**

Ql_xgb_pred **<-**data.frame**(**preds, bin_test**$**biden**)**

#save predictions

write.table**(**Ql_xgb_pred , "Ql_xgb_pred.csv",

sep **=** ",",row.names **=** F, col.names **=** T**)**

#claculate AUC

roc_obj **<-** pROC**::**roc**(**as.vector**(**bin_test**$**biden**)**, preds**)**

auc_xgb **<-** cat**(**"AUC: ", pROC**::**auc**(**roc_obj**))** # AUC Value between 0 to 1

auc_xgb **<-** as.numeric**(**pROC**::**auc**(**roc_obj**))**

auc_xgb

end.gbm **<-** Sys.time**()**

time.xgb **<-** end.gbm **-** srt.gbm

###### Part I - C Forest ######

srt.cf **<-** Sys.time**()**

#prepare the data

train.set **=** sample**(**1**:**90896, size **=** 90896**)**

test.set **=** sample**(**90897**:**113620, size **=** 22724**)**

#Fit a base model

class.task **<-** makeClassifTask**(**data **=** bin_WV, target **=** "biden"**)**

class.lrn **<-** makeLearner**(**"classif.cforest"**)**

WV_class.mod_C **<-** mlr**::**train**(**class.lrn, class.task, subset **=** train.set**)**

WV_class.mod_C

#prepare parameters for model tunning

ps **<-** makeParamSet**(**makeIntegerParam**(**"ntree", lower **=** 1, upper **=** 1000**)**,

makeIntegerParam**(**"mtry", lower **=** 1, upper **=** 3**))**

ctrl **<-** makeTuneControlGrid**(**resolution **=** 10**)**

rdesc **<-** makeResampleDesc**(**"CV", iters **=** 5**)**

#Model tunning (computationaly intensive)

tune.cforest **<-** tuneParams**(**class.lrn, task **=** class.task,

resampling **=** rdesc, par.set **=** ps, **control** **=** ctrl**)**

plotHyperParsEffect**(**generateHyperParsEffectData**(**tune.cforest**)**,

x **=** "ntree", y **=** "mtry", "auc",

plot.type **=** "heatmap"**)**

tune.cforest**$**x

class.lrn.best **<-** setHyperPars**(**makeLearner**(**"classif.cforest"**)**,

ntree **=** tune.cforest**$**x**$**ntree,

mtry **=** tune.cforest**$**x**$**mtry**)**

#Fit the model with best hyperparameters

Ql_cf **<-** mlr**::**train**(**class.lrn.best, class.task, subset **=** train.set**)**

#predictions

task.pred **<-** predict**(**Ql_cf, task **=** class.task, subset **=** test.set**)**

Ql_cif_pred **<-** data.frame**(**task.pred**$**data**)**

head**(**Ql_cif_pred**)**

#Save predictions

write.table**(**Ql_cif_pred, "Ql_cif_pred.csv", sep **=** ","**)**

#calculate AUC

roc_cf **<-** pROC**::**roc**(**as.vector**(**as.numeric**(**Ql_cif_pred**$**truth**))**,

as.numeric**(**Ql_cif_pred**$**response**))**

auc_cf **<-** cat**(**"AUC: ", pROC**::**auc**(**roc_cf**))** # AUC Value between 0 to 1

auc_cf **<-** as.numeric**(**pROC**::**auc**(**roc_cf**))**

auc_cf

end.cf **<-** Sys.time**()**

time.cf **<-** end.cf **-** srt.cf

#####Save summary results####

Model **<-** data.frame**(**"GAM","RMF","CFR","SVM","ANN","XGB"**)**

AUC **<-** data.frame**(**auc_gam, auc_rf, auc_cf, auc_svm, auc_ann, auc_xgb**)**

Time **<-** data.frame**(**time.gam, time.rf, time.cf, time.svm, time.ann, time.xgb**)**

Ql_summary_results_WV **<-** data.frame**(**Ord**=**1**:**6,

Model**=**t**(**Model**)**,

AUC**=**t**(**AUC**)**,

Time**=**t**(**Time**))**

head**(**Ql_summary_results_WV**)**

write.table**(**Ql_summary_results_WV, "Ql_summary_results_WV.csv", sep **=** ",",

row.names **=** F, col.names **=** T**)**

#### PART II - QUANTITATIVE MODELS ######

#Read the original data

data **<-** read.csv**(**"final_pv_vil_week.csv"**)**

#filter out columns with no relevant data (i.e. ID, week, city name, etc)

fil_data **<-** data**[**c**(**6**:**ncol**(**data**))]**

dtest00 **<-** fil_data**[**90897**:**nrow**(**fil_data**)**,**]**

dim**(**dtest00**)**

#split 2009 - 2012 data for train.

#This data still contains zero and non-zero observations

#dtrain0 <- fil_data[1:90896,]

dtrain0 **<-** fil_data**[**1**:**3000,**]**

dim**(**fil_data**)**

dim**(**dtrain0**)**

#In the train data, filter out all those observations with zero values

dtrain **<-** dtrain0 %>% filter**(**logden**>**0**)**

head**(**dtrain**)** # this data contains only non-zero dengue observations

dim**(**dtrain**)**

#Load the binary predictios from the each of the six qualitative models

Ql_pred_GAM **<-** read.csv**(**"Ql_gam_pred.csv"**)**

Ql_pred_RMF **<-** read.csv**(**"Ql_rf_pred.csv"**)**

Ql_pred_CIF **<-** read.csv**(**"Ql_cif_pred.csv"**)**

Ql_pred_CIF **<-** Ql_pred_CIF**[**order**(**Ql_pred_CIF**$**id**)**, **]**

Ql_pred_SVM **<-** read.csv**(**"Ql_svm_pred.csv"**)**

Ql_pred_ANN **<-** read.csv**(**"Ql_ann_pred.csv"**)**

Ql_pred_XGB **<-** read.csv**(**"Ql_xgb_pred.csv"**)**

head**(**Ql_pred_GAM**)**

head**(**Ql_pred_RMF**)**

head**(**Ql_pred_CIF**)**

head**(**Ql_pred_SVM**)**

head**(**Ql_pred_ANN**)**

head**(**Ql_pred_XGB**)**

# bind the columns of the binary predictions with the quantitative test data

#Then filter only the observations predicted as non-zero

dtest_GAM0 **<-** cbind**(**dtest00, Ql_pred_GAM**)**

dtest_GAM0**$**fit **<-** ifelse**(**dtest_GAM0**$**fit**>=**0.5,1,0**)** # classify zero hreshold

dtest_GAM00 **<-** dtest_GAM0 %>% filter**(**fit**==**1**)** #Filter only the predicted as 1

dtest_GAM **<-** dtest_GAM00 **[**c**(**1**:**31**)]** # create the test set for GAM 2nd step HM

head**(**dtest_GAM**)**

dtest_RMF0 **<-** cbind**(**dtest00, Ql_pred_RMF**)**

dtest_RMF00 **<-** dtest_RMF0 %>% filter**(**predictions **==**1**)**

dtest_RMF **<-** dtest_RMF00 **[**c**(**1**:**31**)]**

head**(**dtest_RMF**)**

dtest_ANN0 **<-** cbind**(**dtest00, Ql_pred_ANN**)**

dtest_ANN0**$**fit2 **<-** ifelse**(**dtest_ANN0**$**predict_nnet**>=**0.5,1,0**)**

dtest_ANN00 **<-** dtest_ANN0 %>% filter**(**fit2 **==**1**)**

dtest_ANN **<-** dtest_ANN00 **[**c**(**1**:**31**)]**

head**(**dtest_ANN**)**

dtest_SVM0 **<-** cbind**(**dtest00, Ql_pred_SVM**)**

dtest_SVM0**$**predictedY **<-** ifelse**(**dtest_SVM0**$**predictedY **>=**0.5,1,0**)**

dtest_SVM00 **<-** dtest_SVM0 %>% filter**(**predictedY**==**1**)**

dtest_SVR **<-** dtest_SVM00 **[**c**(**1**:**31**)]**

head**(**dtest_SVR**)**

dtest_XGB0 **<-** cbind**(**dtest00, Ql_pred_XGB**)**

dtest_XGB0**$**preds **<-** ifelse**(**dtest_XGB0**$**preds **>=**0.5,1,0**)**

dtest_XGB00 **<-** dtest_XGB0 %>% filter**(**preds**==**1**)**

dtest_XGB **<-** dtest_XGB00 **[**c**(**1**:**31**)]**

head**(**dtest_XGB**)**

dtest_CIF0 **<-** cbind**(**dtest00, Ql_pred_CIF**)**

dtest_CIF0**$**response **<-** ifelse**(**dtest_CIF0**$**response **>=**0.5,1,0**)**

dtest_CIF00 **<-** dtest_CIF0 %>% filter**(**response**==**1**)**

dtest_CIF **<-** dtest_CIF00 **[**c**(**1**:**31**)]**

head**(**dtest_CIF**)**

nrow**(**dtest_ANN**)**

nrow**(**dtest_CIF**)**

nrow**(**dtest_RMF**)**

nrow**(**dtest_XGB**)**

nrow**(**dtest_GAM**)**

nrow**(**dtest_SVR**)**

###### Part II - GAM ######

srt.gam **<-** Sys.time**()**

#fit a base model

WV_GAM_HY **<-** gam**(**logden **~** **(**Edu**)+(**Ind**)+(**Hig**)+**s**(**tmin_L16**)+**

**(**tmean_L17**)+(**uw_L5**)+(**Hea**)+(**Com**)+**

**(**Vhi**)+(**tmax_L17**)+(**ndvi_L0**)+(**vw_L11**)+**

**(**Cem**)+(**Tra**)+(**RND**)+(**l_sgpm_L6**)+(**rh_L4**)+**

**(**Mil**)+(**Inf.**)+(**FloodRisk**)+(**Gov**)+(**Vlo**)+**

**(**Low**)+(**Agr**)+(**Gra**)+(**For**)+(**Wat**)+(**Ope**)+(**Par**)**,

data **=** dtrain, family **=** gaussian**(**link**=**identity**))**

#prepare for model tunning

sp **<-** WV_GAM_HY**$**sp

tuning.scale**<-**c**(**1e-5,1e-4,1e-3,1e-2,1e-1,1e0,1e1,1e2,1e3,1e4,1e5**)**

scale.exponent**<-**log10**(**tuning.scale**)**

n.tuning**<-**length**(**tuning.scale**)**

edf**<-**rep**(NA**,n.tuning**)**

min2ll**<-**rep**(NA**,n.tuning**)**

aic**<-**rep**(NA**,n.tuning**)**

bic**<-**rep**(NA**,n.tuning**)**

#Tune the model

**for** **(**i **in** 1**:**n.tuning**)** **{**

WV_GAM_HY **<-**gam**(**logden **~** **(**Edu**)+(**Ind**)+(**Hig**)+**s**(**tmin_L16**)+**

**(**tmean_L17**)+(**uw_L5**)+(**Hea**)+(**Com**)+**

**(**Vhi**)+(**tmax_L17**)+(**ndvi_L0**)+(**vw_L11**)+**

**(**Cem**)+(**Tra**)+(**RND**)+(**l_sgpm_L6**)+(**rh_L4**)+**

**(**Mil**)+(**Inf.**)+(**FloodRisk**)+(**Gov**)+(**Vlo**)+**

**(**Low**)+(**Agr**)+(**Gra**)+(**For**)+(**Wat**)+(**Ope**)+(**Par**)**,

sp**=**tuning.scale**[**i**]***sp,

data **=** dtrain,

family **=** gaussian**(**link**=**identity**))**

min2ll**[**i**]<--**2*****logLik**(**WV_GAM_HY**)**

edf**[**i**]<-**sum**(**WV_GAM_HY**$**edf**)+**1

aic**[**i**]<-**AIC**(**WV_GAM_HY**)**

bic**[**i**]<-**BIC**(**WV_GAM_HY**)**

**}**

#visualizations

par**(**mfrow**=**c**(**2,2**))**

plot**(**scale.exponent,min2ll,type**=**"b",main**=**"2 log likelihood"**)**

plot**(**scale.exponent,edf,ylim**=**c**(**0,70**)**,type**=**"b",

main**=**"effective number of parameters"**)**

plot**(**scale.exponent,aic,type**=**"b",main**=**"AIC"**)**

plot**(**scale.exponent,bic,type**=**"b",main**=**"BIC"**)**

min.bic**<-**1e100

opt.tuning.scale**<-NULL**

**for** **(**i **in** 1**:**n.tuning**)** **{**

**if** **(**bic**[**i**]<**min.bic**)** **{**

min.bic**<-**bic**[**i**]**

opt.tuning.scale**<-**tuning.scale**[**i**]**

**}**

**}**

opt.sp**<-**opt.tuning.scale*****sp

#Fit the model with best parameters

Qt_GAM **<-**gam**(**logden **~** **(**Edu**)+(**Ind**)+(**Hig**)+**s**(**tmin_L16**)+**

**(**tmean_L17**)+(**uw_L5**)+(**Hea**)+(**Com**)+**

**(**Vhi**)+(**tmax_L17**)+(**ndvi_L0**)+(**vw_L11**)+**

**(**Cem**)+(**Tra**)+(**RND**)+(**l_sgpm_L6**)+(**rh_L4**)+**

**(**Mil**)+(**Inf.**)+(**FloodRisk**)+(**Gov**)+(**Vlo**)+**

**(**Low**)+(**Agr**)+(**Gra**)+(**For**)+(**Wat**)+(**Ope**)+(**Par**)**,

sp**=**opt.sp,

data **=** dtrain,

family **=** gaussian**(**link**=**identity**))**

summary**(**Qt_GAM**)**

#predictions

Qt_gam_pred **<-** data.frame**(**predict**(**Qt_GAM, newdata**=**dtest_GAM,

type**='response'**, se**=**T**))**

Qt_gam_pred **<-** data.frame**(**pred **=** Qt_gam_pred**$**fit, logden **=** dtest_GAM**$**logden**)**

head**(**Qt_gam_pred**)**

#save predictions

write.table**(**Qt_gam_pred, "Qt_gam_pred.csv",

col.names **=** T, row.names **=** F, sep **=** ","**)**

#calculate metrics

error.gam **<-** rmse**(**Qt_gam_pred**$**pred, Qt_gam_pred**$**logden**)**

R_Sqr.gam **<-** cor**(**Qt_gam_pred**$**pred, Qt_gam_pred**$**logden**)^**2

R_Sqr.gam

error.gam

end.gam **<-** Sys.time**()**

time.gam **<-** end.gam **-** srt.gam

###### Part II - Random Forest ######

srt.rf **<-** Sys.time**()**

#set the formula

formula **<-** logden **~**Agr**+**Gra**+**For**+**Wat**+**Ope**+**Par**+**Edu**+**Hea**+**Cem**+**Mil**+**Gov**+**

Ind**+**Com**+**Tra**+**Inf.**+**Vlo**+**Low**+**Med**+**Hig**+**Vhi**+**RND**+**FloodRisk**+**tmin_L16**+**

tmax_L17**+**tmean_L17**+**ndvi_L0**+**l_sgpm_L6**+**uw_L5**+**vw_L11**+**rh_L4

#prepare the tunning grid

grid.rf **<-** expand.grid**(**

mtry **=** seq**(**1, 3, by **=** 1**)**,

ntree **=** seq**(**100,1000, by**=**100**)**,

node_size **=** seq**(**2, 10, by **=** 2**)**,

sampe_size **=** c**(**0.80,0.9,1.0**)**,

OOB_RMSE **=** 0**)**

# total number of combinations

nrow**(**grid.rf**)**

#model tunning

**for(**i **in** 1**:**nrow**(**grid.rf**))** **{**

# train model

mod **<-** ranger**(**

formula **=** formula ,

data **=** dtrain,

num.trees **=** grid.rf**$**ntree**[**i**]**,

mtry **=** grid.rf**$**mtry**[**i**]**,

min.node.size **=** grid.rf**$**node_size**[**i**]**,

sample.fraction **=** grid.rf**$**sampe_size**[**i**]**

**)**

# add OOB error to grid

grid.rf**$**OOB_RMSE**[**i**]** **<-**sqrt**(**mod**$**prediction.error**)}**

#check the outputs and select best hyperparameters

grid.rf %>%

dplyr**::**arrange**(**OOB_RMSE**)** %>%

head**(**10**)**

#Fit the model with best hyperparameters

Qt_RF **<-** ranger**(**formula,num.trees **=** 1000, mtry **=**3,

min.node.size **=** 3, sample.fraction **=** 1.0,

data **=** dtrain, verbose **=** T**)**

#Make predictions

rang_pred **<-** predict**(**Qt_RF, dtest_RMF**)**

head**(**rang_pred**)**

Qt_rf_pred **<-** data.frame**(**predicted **=** predictions**(**rang_pred,WV_RF_HY**)**,

logden **=** dtest_RMF**$**logden**)**

head**(**Qt_rf_pred**)**

#save predictions

write.table**(**Qt_rf_pred, "Qt_rf_pred.csv", col.names **=** T,

row.names **=** F, sep **=** ","**)**

#calculate metrics

error.rf **<-** rmse**(**Qt_rf_pred**$**predicted, Qt_rf_pred**$**logden**)**

R_Sqr.rf **<-** cor**(**Qt_rf_pred**$**predicted, Qt_rf_pred**$**logden**)^**2

R_Sqr.rf

error.rf

end.rf **<-** Sys.time**()**

time.rf **<-** end.rf **-** srt.rf

####### Part II - Neural Networks #######

srt.ann **<-** Sys.time**()**

#tune the model

WV_ANN_HY **<-** tune.nnet**(**logden **~** .,

data**=**dtrain,

size**=**1**:**10, linout**=TRUE**, skip**=**T,

MaxNWts**=**100000, trace**=**T,

maxit**=**1000**)**

summary**(**WV_ANN_HY**)**

#Fit the model with best hyperparameters

Qt_ANN **<-** nnet**(**logden **~** .,

data**=**dtrain,

size**=**2, linout**=TRUE**, skip**=**T,

MaxNWts**=**100000, trace**=**T,

maxit**=**1000**)**

#predict

predict_nnet **=** predict**(**Qt_ANN, newdata**=**dtest_ANN**)**

Qt_ann_pred **=** data.frame**(**predictions **=** predict_nnet,

logden **=** dtest_ANN**$**logden**)**

head**(**Qt_ann_pred**)**

#Save predictions

write.table**(**Qt_ann_pred, "Qt_ann_pred.csv", col.names **=** T,

row.names **=** F, sep **=** ","**)**

#calculate meterics

error.ann **<-** rmse**(**Qt_ann_pred**$**predictions, Qt_ann_pred**$**logden**)**

R_Sqr.ann **<-** cor**(**Qt_ann_pred**$**logden, Qt_ann_pred**$**predictions**)^**2

R_Sqr.ann

error.ann

end.ann **<-** Sys.time**()**

time.ann **<-** end.ann **-** srt.ann

########Part II - SVR #######################

srt.svr **<-** Sys.time**()**

#Tune the model

WV_SVR_HY **<-** tune.svm**(**logden**~**.

, data **=** dtrain, kernel **=** "radial",

degree**=**seq**(**0,3.5, by **=** 0.1**)**, coef0**=**c**(**0.001,0.01**)**,

verbose **=** T, trace **=**T**)**

summary**(**WV_SVR_HY**)**

#select the best model

Qt_SVR **<-** WV_SVR_HY**$**best.model

#predict

predictedY **<-** predict**(**Qt_SVR, dtest_SVR**)**

Qt_svr_pred **<-** data.frame**(**prediction **=** predictedY,

logden **=** dtest_SVR**$**logden**)**

head**(**Qt_svr_pred**)**

#Save predictions

write.table**(**Qt_svr_pred, "Qt_svr_pred.csv", col.names **=** T,

row.names **=** F, sep **=** ","**)**

#claculate metrics

error.svr **<-** rmse**(**Qt_svr_pred**$**prediction, Qt_svr_pred**$**logden**)** # 3.157061

R_Sqr.svr **<-** cor**(**Qt_svr_pred**$**prediction, Qt_svr_pred**$**logden**)^**2

R_Sqr.svr

error.svr

end.svr **<-** Sys.time**()**

time.svr **<-** end.svr **-** srt.svr

########## Part II - XGBM #############

srt.gbm **<-** Sys.time**()**

#prepare data

traind **<-** xgb.DMatrix**(**

data **=** data.matrix**(**subset**(**dtrain, select **=** **-**logden**))**,

label **=** data.matrix**(**dtrain**$**logden**))**

#tune the model

xgb **<-** xgb.cv**(**data **=** traind,

eta **=** 0.0001,

max_depth **=** 50,

nround **=** 6000,

subsample **=** 0.5,

colsample_bytree **=** 0.5,

objective **=** "reg:squarederror",

nthread **=** 8,

nfold **=** 5,

early_stopping_rounds **=** 50,

eval_metric **=** **'rmse')**

#select the best model

best.gbm **<-** xgb**$**best_iteration

#fit the model with best hyperparameters

Qt_XGB **<-** xgboost**(**data **=** traind,

eta **=** 0.0001,

max_depth **=** 50,

nround **=** best.gbm,

subsample **=** 0.5,

colsample_bytree **=** 0.5,

objective **=** "reg:squarederror",

nthread **=** 8,

eval_metric **=** **'rmse')**

#predictions

preds **<-** predict**(**Qt_XGB,

data.matrix**(**subset**(**dtest_XGB, select **=** **-**logden**)))**

Qt_xgb_pred **<-**data.frame**(**prediction **=** preds,

logden **=** dtest_XGB**$**logden**)**

#Save predictions

write.table**(**Qt_xgb_pred, "Qt_xgb_pred.csv", col.names **=** T,

row.names **=** F, sep **=** ","**)**

#calculate metrics

error.gbm **<-** rmse**(**Qt_xgb_pred**$**prediction, Qt_xgb_pred**$**logden**)**

R_Sqr.gbm **<-** cor**(**Qt_xgb_pred**$**prediction, Qt_xgb_pred**$**logden**)^**2

error.gbm

R_Sqr.gbm

end.gbm **<-** Sys.time**()**

time.gbm **<-** end.gbm **-** srt.gbm

########### Part II - C forest ###################

srt.cf **<-** Sys.time**()**

#merge train and test data since they are stored in different objects

join **<-** rbind**(**dtrain, dtest_CIF**)**

dim**(**join**)**

nrow**(**dtrain**)**

#Set the range for train and test

train.set **<-** sample**(**1**:**25979, size **=** 25979**)**

test.set **<-** sample**(**25980**:**nrow**(**join**))**

#Fit a base model

regr.task **<-** makeRegrTask**(**data **=** join, target **=** "logden"**)**

regr.lrn **<-** makeLearner**(**"regr.cforest"**)**

regr.mod **<-** mlr**::**train**(**regr.lrn, regr.task, subset **=** train.set**)**

regr.mod

#prepate model tunning

ps **<-** makeParamSet**(**makeIntegerParam**(**"ntree", lower **=** 1, upper **=** 1000**)**,

makeIntegerParam**(**"mtry", lower **=** 1, upper **=** 2**))**

ctrl **<-** makeTuneControlGrid**(**resolution **=** 10**)**

rdesc **<-** makeResampleDesc**(**"CV", iters **=** 5**)**

#tune the model

tune.cforest **<-** tuneParams**(**regr.lrn, task **=** regr.task,

resampling **=** rdesc, par.set **=** ps,

**control** **=** ctrl**)**

tune.cforest**$**x

regr.lrn.best **<-** setHyperPars**(**makeLearner**(**"regr.cforest"**)**,

ntree **=** tune.cforest**$**x**$**ntree,

mtry **=** tune.cforest**$**x**$**mtry**)**

#Fit the model with best hyperparameters

Qt_CIF **=** mlr**::**train**(**regr.lrn.best, regr.task, subset **=** train.set**)**

#predictions

Pred **=** predict**(**Qt_CIF, task **=** regr.task, subset **=** test.set**)**

Qt_cf_pred **<-** data.frame**(**prediction **=** Pred**$**data**$**response,

logden **=** Pred**$**data**$**truth**)**

#Save predictions

write.table**(**Qt_cf_pred, "Qt_cf_pred.csv",

col.names **=** T, row.names **=** F, sep **=** ","**)**

#Calculate metrics

error.cf **<-** rmse**(**Qt_cf_pred**$**prediction, Qt_cf_pred**$**logden**)**

R_Sqr.cf **<-** cor**(**Qt_cf_pred**$**prediction, Qt_cf_pred**$**logden**)^**2

R_Sqr.cf

error.cf

end.cf **<-** Sys.time**()**

time.cf **<-** end.cf **-** srt.cf

######Save summary results of part II#########

Model **<-** data.frame**(**"GAM","RF","CF","SVR","ANN","XGBM"**)**

n_train **<-** data.frame**(**nrow**(**dtrain**)**,nrow**(**dtrain**)**,nrow**(**dtrain**)**,

nrow**(**dtrain**)**,nrow**(**dtrain**)**,nrow**(**dtrain**))**

n_test **<-** data.frame**(**nrow**(**dtest_GAM**)**,nrow**(**dtest_RMF**)**,

nrow**(**dtest_CIF**)**,nrow**(**dtest_SVR**)**,

nrow**(**dtest_ANN**)**,nrow**(**dtest_XGB**))**

RMSE **<-** data.frame**(**error.gam, error.rf, error.cf,

error.svr, error.ann, error.gbm**)**

R_Sqr **<-** data.frame**(**R_Sqr.gam, R_Sqr.rf, R_Sqr.cf,

R_Sqr.svr, R_Sqr.ann, R_Sqr.gbm**)**

Time **<-** data.frame**(**time.gam, time.rf, time.cf, time.svm, time.ann, time.gbm**)**

Qt_summary_results_WV **<-** data.frame**(**Ord**=**1**:**6, Model**=**t**(**Model**)**,

n_train**=**t**(**n_train**)**,n_test**=**t**(**n_test**)**,

RMSE**=**t**(**RMSE**)**,R_Sqr**=**t**(**R_Sqr**)**,Time**=**t**(**Time**))**

head**(**Qt_summary_results_WV**)**

write.table**(**Qt_summary_results_WV, "Qt_summary_results_WV.csv",

sep **=** ",", col.names **=** T, row.names **=** F**)**
