# Supplementary material for: Hybrid Machine Learning Approach to Zero-Inflated Data Improves Accuracy of Dengue Prediction
Source: PLoS Negl Trop Dis. 2024 Oct 21;18(10):e0012599. doi: 10.1371/journal.pntd.0012599 (PMC11527386; doi:10.1371/journal.pntd.0012599)
Supplement: Attachment — Submitted filename: Review for PLOS NTD 2023.docx [file pntd.0012599.s013.docx]

Review for PLOS NTD 2023

The paper, “Hybrid Machine Learning Approach to Zero-Inflated Data Improves Accuracy of

Dengue Prediction”, takes a thorough approach to looking at how models are able to predict disease cases at different scales. The authors consider 3 spatial scales and 2 time scales where the finer resolution scales are aggregated to get the lower resolution scales. They compare a set of modeling algorithms and consider how the best algorithm may also vary with scale. Finally, they conclude that a two-step process is most appropriate for the finest scale (week-village).

I think the authors have done a good job, and I appreciate the hybrid approach that is sort of like a forecasting analog to hurdle models used in parametric statistics for zero-inflated data. I agree that this paper could have general influence beyond the immediate forecast of dengue that is considered here. The authors did a good job of explaining the complexities of the analysis, but I found a few areas where things needed further clarification. I also did not notice any mention of the code being available, which I consider to be a crucial aspect of this paper as it is introducing new approaches that the authors want other to use and because they find support for a less widely used method (CIF).

Major items

There are several aspects of the methods that need to be explained further. Many of these are in the minor comments below, but there are a couple key items. 1) It would be quite helpful to see a table of the actual variables used in each model. Table S2 seems to show the remote sensed variables that were used at certain lags, but in Table S4 there is reference to 30 variables being used. I have no way to get from the 8 lagged variables at each time frame to 30 variables even when thinking about population density, etc. Maybe I missed this, but it needs to be more clearly shown. Another aspect that should be clarified is the training-test split. In some areas years are referred to (2009-2012 training and 2013 test) and elsewhere 80% and 20%. Do these correspond directly? Maybe this is another case where an additional element, like a flowchart, could help clarify. Finally, it would be helpful to see at least an example of the actual dengue data entering the model. Are zeros clustered in space or time? How seasonal are they? Do they disappear as soon as aggregated in either space or time? This could be another set of supplementary figures, the authors would know the best way to convey the data patterns leading to their decisions about zero-inflation. And to see how log-transformation (L156) altered the distribution.

Unless I missed it, the authors don’t explain if the spatial data is averaged to the village/city/region polygons or values are taken at the centroid. I assumed the former based on other text, but couldn’t find a statement. This is also a case where code is helpful for clarity.

The authors present 3 table/figures using merged villages, but these are not explained in the results and only mentioned briefly in the discussion. If they are included I think they should be explained/explored more fully.

One approach for dealing with zeros in presence-absence data is to balance the training by either upsampling or downsampling one of the categories. It is also good practice to balance the test set, which the authors also didn’t consider. This seems like it could be relevant to the discussion in lines 463-494.

Minor comments

L159 Variables are listed as 0.1 degree resolution in Table S1

L203 Was the cross-correlation analysis only using the training data or the whole dataset?

L208 weeks is indicated twice in this description, which is confusing. I think the second should be months. Why were these number of lags chosen?

L213 Meaning that the weekly models used the 8 weekly variable lags in Table S2 and monthly models used the 8 monthly variable lags in Table S2?

L237 I didn’t quite follow the use of only environmental variables. Why not continue to include the static variables while changing the environmental variables? Otherwise you are changing how the models can account for the influence of the static variables, which may change the information being loaded on the environmental variables. Or did I misunderstand?

L286 Is this because the data was 30% non-zero occurrence? Make explicit.

L246 Include a table or flowchart in the supplement to show the data usage at each scale? See comment for Table 1 below

L246 Training and test could be more clearly explained. In all cases the 80% 20% split was used and this was based on the time alone (with 2013 being the test)? Separate explanation for hybrid is confusing in this sense.

L301 Why is RF not mentioned for week-region despite lower RMSE?

L301 CIF seems essentially indistinguishable for month-village, which would follow the pattern of CIF being strong at all sub-region scales.

Table 1, I assume the values in parentheses representing the number of zeros are only relevant for week-village, how many zeros were there at the other, aggregated scales?

L356 Not clear if the hybrid of AUC and S-Ra2 can be interpreted as explaining 77% of variance. Seems like a key point is the improvement (from the zero-inflated dataset) in S-Ra2 for all models except GAM

L431 Is the goal to get the true number of cases or is the number of cases needing medical care actually a more useful outcome? Is there any reason to think the variables are actually predicting number of cases needing medical care rather than true number of cases? I.e. are there seasonal differences in severity of symptoms or spatial differences in underlying health outcomes that could be explored?

L464 “six-fold” is based on the S-Ra2 (which seems to be a slight overestimate) or HAI (which seems to be comparison of non-comparable values)?

L521 “Supplemental to the R package…” seems to be incomplete thought.

Supplementary information – S3 Random forest, was the hyper-parameter tuning done in an automated fashion via ‘ranger’?
