# Supplementary material for: Hybrid Machine Learning Approach to Zero-Inflated Data Improves Accuracy of Dengue Prediction
Source: PLoS Negl Trop Dis. 2024 Oct 21;18(10):e0012599. doi: 10.1371/journal.pntd.0012599 (PMC11527386; doi:10.1371/journal.pntd.0012599)
Supplement: Attachment — Submitted filename: PLOS NTDs_Response to the review.docx [file pntd.0012599.s014.docx]

Dear Editor,

Attached herewith is the revised version of our manuscript entitled: **Hybrid Machine Learning Approach to Zero-Inflated Data Improves Accuracy of Dengue Prediction.** We modified the manuscript following the suggestions made by the Editor and the two Reviewers. We answered and explained point by point the issues, comments, and suggestions made by the Editor and Reviewers. The manuscript has been revised accordingly with track changes and highlights for your guidance. Since there are numerous comments, each comment was provided with codes. The line numbers indicated in the table below correspond to the line numbering in the manuscript file without track changes. Since the questions and comments from the Editor and Reviewers differed, we have created within the table specific sections for the Editor and Reviewers to facilitate locating the responses.

We are very thankful to the Editor and Reviewers because their valuable insights made our manuscript better. We are hoping that our revisions will merit the approval for publication in your prestigious journal.

Thank you!

| Code | Question | Answer |
| --- | --- | --- |
| Editor | | |
| ED-01 | I think the authors have done a good job, and I appreciate the hybrid approach that is sort of like a forecasting analog to hurdle models used in parametric statistics for zero-inflated data. I agree that this paper could have general influence beyond the immediate forecast of dengue that is considered here. The authors did a good job of explaining the complexities of the analysis, but I found a few areas where things needed further clarification. | Thank you for your thoughtful and encouraging comments on our work. We sincerely appreciate your positive assessment of our hybrid approach and its potential impact beyond the specific context of dengue forecasting.  We acknowledge your feedback regarding areas where further clarification is needed. We are committed to enhancing the clarity and completeness of our analysis. Your insights are invaluable, and we have addressed the identified points to ensure that our paper meets the highest standards of clarity and rigor. |
| ED-02 | I also did not notice any mention of the code being available, which I consider to be a crucial aspect of this paper as it is introducing new approaches that the authors want other to use and because they find support for a less widely used method (CIF). | Thank you for your technical comment. To demonstrate how the analyses were conducted in this study we have added our code for the implementation of the hybrid approach in the supplementary material.  Please see Code S7.  We have also added the conceptual figure for the hybrid model. To make the less widely used modeling approach applicable to a wide range of scientists, we believe it is important to provide a conceptual figure that helps them understand the model as well as the code we have provided.  Please see Fig. 1  We have also added the code in GitHub accessible through the link: <https://github.com/Micanaldo/Hybrid-Machine-Learning-Model> |
| ED-03 | Major items  There are several aspects of the methods that need to be explained further. Many of these are in the minor comments below, but there are a couple key items. 1) It would be quite helpful to see a table of the actual variables used in each model. Table S2 seems to show the remote sensed variables that were used at certain lags, but in Table S4 there is reference to 30 variables being used. I have no way to get from the 8 lagged variables at each time frame to 30 variables even when thinking about population density, etc. Maybe I missed this, but it needs to be more clearly shown. | The Authors apologize for the confusion generated by not explicitly stating the names of all 30 variables. We would like to clarify that all spatiotemporal models at the village and city scales used a total of 30 variables. From these, 8 are climatic and 22 are landscape variables. The former Table S2 only listed climatic variables because were applied to lag analysis with dengue incidence as they varied in time. The remaining 22 variables were static landscapes such as flood risk, roads, and land use. Therefore, they were not included in the former Table S2. Models at a regional scale and the individual village models only used the 8 climatic variables. Static landscape variables were excluded from these models because no relevant contribution would be expected since these have no variance in time within individual villages or at the regional scale.  For more clarity for the readers, we have added the list of all variables used for each model in the study as supplementary material.  Please see Table S5 |
| ED-04 | Another aspect that should be clarified is the training-test split. In some areas years are referred to (2009-2012 training and 2013 test) and elsewhere 80% and 20%. Do these correspond directly? Maybe this is another case where an additional element, like a flowchart, could help clarify. | We apologize for the confusion generated by our statement. Yes, they mean the same. In the hybrid approach, we used the same splitting method as in the qualitative and quantitative models (2009 – 2012 for train and 2013 for testing). Data from 2009 – 2012 corresponds to 90,896 out of 113,620 observations (80%) while 2013 corresponds to 22,724 out of 113,620 observations (20%). Because of it was confusing, we edited the text about the splitting method for the hybrid approach for consistency in the manuscript.  Please see Line 315. |
| ED-05 | Finally, it would be helpful to see at least an example of the actual dengue data entering the model. Are zeros clustered in space or time? How seasonal are they? Do they disappear as soon as aggregated in either space or time? This could be another set of supplementary figures, the authors would know the best way to convey the data patterns leading to their decisions about zero-inflation. And to see how log-transformation (L156) altered the distribution. | Thank you. We have generated histograms of data distribution for each spatiotemporal resolution indicating the number of zeros after data was aggregated. This file is added as supplementary material (Figure S11). Figure S11 shows a higher reduction of zero values when data is aggregated in the spatial dimension (from village to city or region) than temporal aggregation (from week to month).  Please see Figure S11.  We have also added these insights in the discussion section.  Please see Lines 452 – 456.  The log transformation compressed the extreme values, thus reducing the number of outliers.  Please see Figure S11 (A) and (B). |
| ED-06 | Unless I missed it, the authors don’t explain if the spatial data is averaged to the village/city/region polygons or values are taken at the centroid. I assumed the former based on other text, but couldn’t find a statement. This is also a case where code is helpful for clarity. | Thank you. All climatic variables were available in raster format (pixels). Because the boundaries of a village did not coincide with the boundaries of the pixels, the value of one variable in one village was the average from all the pixels overlapping with that village. However, we did not use the common mean value for averaging the pixels, instead, we used the spatially weighted average. Please, consider the figure below as a demonstration example.    Let in this example the black contour shape be the village and the red squares be the pixels of a one-week temperature raster file. To calculate the weighted average of temperature in this village for this week, we summed the values of temperature in each pixel multiplied by the percentage of the village intersecting each pixel as below.    This would result in a temperature of 23.32ºC while if using the common mean value, the temperature would be 23.23ºC. The differences are because the late temperature ignored the contribution of each pixel to the village area, thus, biased.  This approach despite being time-consuming and computationally intensive, allows for fairness in the spatial extraction of data from pixels to village polygons.  To make this approach clear to the readers, we have added this explanation to the methods section.  Please see Lines 214 – 220. |
| ED-07 | The authors present 3 table/figures using merged villages, but these are not explained in the results and only mentioned briefly in the discussion. If they are included I think they should be explained/explored more fully. | Thank you.  We have added the description of the results with merged villages in the manuscript.  Please see Lines 380 – 389. |
| ED-08 | One approach for dealing with zeros in presence-absence data is to balance the training by either upsampling or downsampling one of the categories. It is also good practice to balance the test set, which the authors also didn’t consider. This seems like it could be relevant to the discussion in lines 463-494. | Yes. Upsampling and downsampling are common approaches to dealing with imbalanced data in qualitative prediction. However, the model's accuracy can be significantly influenced by the choice of sampling sizes when utilizing these methods. Upsampling involves randomly duplicating examples from the minority class to balance the class distribution, which may lead to overfitting due to the repetition of similar instances. Conversely, downsampling reduces the number of instances in the majority class, which may lead to the loss of valuable information and reduced model performance. These variations in sampling sizes can introduce uncertainties regarding the stability of the model, as the model's predictive capability may become overly reliant on the specific instances selected during the sampling process ( <https://doi.org/10.1186/s12859-022-04761-4> ) . In our qualitative models, we observed fair model accuracy and therefore did not find it necessary to apply upsampling or downsampling. However, it's important to note that upsampling, downsampling, Synthetic Minority Over-sampling Technique (SMOTE), or other similar techniques can be integrated into the first step of our hybrid model if one finds these techniques to substantially improve model accuracy for their specific modeling scenarios. The hybrid approach framework is designed to be generalizable and flexible, allowing for the incorporation of various techniques to meet the specific needs and characteristics of the data. This adaptability ensures that the modeling process can be tailored to the unique circumstances of the dataset.  We have included these observations in the discussion (please see Lines 563 – 571) sections. |
| ED-09 | Minor comments  L159 Variables are listed as 0.1 degree resolution in Table S1 | We replaced the text indicating the spatial resolution in kilometers.  Please see Table S2. |
| ED-10 | L203 Was the cross-correlation analysis only using the training data or the whole dataset? | We employed cross-correlation analysis with the entire dataset before splitting train and test subsets. We have edited the sentence for clarity. The new text is:  “A cross-correlation analysis using the entire data from 2009 – 2013 was performed between the weekly or monthly temporal variations in environmental factors (i.e., minimum temperature, mean temperature, maximum temperature, precipitation, northward wind, eastward wind, RH, and NDVI) and dengue incidence.”  Please see Line 233 |
| ED-11 | L208 weeks is indicated twice in this description, which is confusing. I think the second should be months. Why were these number of lags chosen? | Yes. The second “weeks” should be “months”. We have resolved the mistyping.  Please see Line 241  Dengue incidence may not immediately respond to changes in climatic or environmental factors. Exploring different lag times allows the model to capture delayed effects and understand the temporal relationships between predictors and dengue incidence. By incorporating lagged predictors based on correlation analysis, the machine learning model can potentially improve its predictive performance. Lagged features account for the time delays in the impact of climatic factors on dengue incidence, providing a more accurate representation of the underlying dynamics. The selection of lag time points was not predetermined initially. We conducted a correlation analysis between dengue incidence and climatic factors, starting from lag 0 and extending onwards. The objective was to identify the time point at which the correlation exhibited the highest magnitude while maintaining statistical significance in our tests. Upon plotting a vector of Pearson correlations, we discerned the week or month with the highest correlation, leading to its selection as the optimal lag time. The lag number of weeks or months (best-lag time) is based on the highest Pearson correlation coefficient and whether the p-value is ≤0.05. We have added the explanation to the manuscript.  Please see Lines 238 – 246 |
| ED-12 | L213 Meaning that the weekly models used the 8 weekly variable lags in Table S2 and monthly models used the 8 monthly variable lags in Table S2? | Yes, that is correct.  Please see Table S4 and Table S5 |
| ED-13 | L237 I didn’t quite follow the use of only environmental variables. Why not continue to include the static variables while changing the environmental variables? Otherwise you are changing how the models can account for the influence of the static variables, which may change the information being loaded on the environmental variables. Or did I misunderstand? | The Authors considered removing the static variables from the individual village temporal models a reasonable approach. Firstly, the datasets used for the spatiotemporal models included several locations which had different land use compositions (spatial heterogeneity) and also different climatic characteristics over time (temporal heterogeneity). In this case, the model would account for existing differences in both space and time among villages. In the individual village models, the situation is quite different. While climatic factors change in time (temporal heterogeneity), in each village the land use did not change in time. Since these models were purely temporal, the assumption is that features like land use, which are static within each location and do not change over time, might not be influential in explaining variations in dengue incidence within a specific location. Including them in the temporal model might introduce unnecessary complexity without providing additional information. In the view of the Authors, the inclusion of the static features in the individual village temporal models would require a different modeling algorithm that can account for interaction terms (i.e. <https://doi.org/10.1016/j.scitotenv.2021.148406>), which is different from the model approach in this study. We have added this explanation to the methods section.  Please see Lines 274 – 278. |
| ED-14 | L286 Is this because the data was 30% non-zero occurrence? Make explicit. | Yes, that is correct. For clarity, we have edited the manuscript.  Please see Lines 329 – 330. |
| ED-15 | L246 Include a table or flowchart in the supplement to show the data usage at each scale? See comment for Table 1 below | Thank you for the suggestion. We have added a table in the supplementary material showing the data used at each model.  Please see Table S5. |
| ED-16 | L246 Training and test could be more clearly explained. In all cases the 80% 20% split was used and this was based on the time alone (with 2013 being the test)? Separate explanation for hybrid is confusing in this sense. | We apologize for the confusion generated by our statement. In the hybrid approach, we used the same splitting method as in the qualitative and quantitative models (2009 – 2012 for train and 2013 for testing). Data from 2009 – 2012 corresponds to 90,896 out of 113,620 observations (80%) while 2013 corresponds to 22,724 out of 113,620 observations (20%). Because of it was confusing, we edited the text about the splitting method for the hybrid approach for consistency in the manuscript.  Please see Line 315. |
| ED-17 | L301 Why is RF not mentioned for week-region despite lower RMSE? | Thank you for pointing the omission. We have added the following sentence on the manuscript:  “Although RF exhibited a lower capacity to elucidate the variance in dengue within the week-village model, it attained the highest level of predictive accuracy at this resolution (RMSE = 0.019).”  Please see Lines 347 – 349. |
| ED-18 | L301 CIF seems essentially indistinguishable for month-village, which would follow the pattern of CIF being strong at all sub-region scales. | Yes. In this resolution, CIF yields very close results to RF. We have bolded the RMSE value of CIF since was equal to RF. The other metrics are left as before since differ notably with decimal places.  Please see Table 1. |
| ED-19 | Table 1, I assume the values in parentheses representing the number of zeros are only relevant for week-village, how many zeros were there at the other, aggregated scales? | We have added the number of zeros for the other datasets.  Please see columns 2 and 3 in the Table 1. |
| ED-20 | L356 Not clear if the hybrid of AUC and S-Ra2 can be interpreted as explaining 77% of variance. Seems like a key point is the improvement (from the zero-inflated dataset) in S-Ra2 for all models except GAM | Since the AUC only tells the ability of the model to distinguish one class from the other, we do not recommend the combined HAI to be interpreted as 77% of the explained variance. The % of the variance can be explained by S-Ra2. We think it would be correct to make this interpretation only for the second step of the hybrid model, where the quantitative model was applied. For example, it is correct to say that the second step of the hybrid model was able to explain the variance in dengue incidence by 71.6%. Based on the above stated, the Authors have decided to rephrase the previous L356 to:  “Although the quantitative model could only explain 11.6% of the variations in dengue incidence, the second step of the hybrid model could explain approximately 71.6% of the variations in dengue incidence in villages expected to be dengue positive from the first step.”  Please see Lines 420 – 422. |
| ED-21 | L431 Is the goal to get the true number of cases or is the number of cases needing medical care actually a more useful outcome? Is there any reason to think the variables are actually predicting number of cases needing medical care rather than true number of cases? I.e. are there seasonal differences in severity of symptoms or spatial differences in underlying health outcomes that could be explored? | Thank you for your insightful comment. Since the dengue cases utilized in this study were recorded from a passive surveillance system, our predictions are for the number of cases seeking medical care. We have edited the incongruent statement in the manuscript. Furthermore, we have added that assuming that disease case counts are often limited to those reported from passive surveillance systems, modeling approaches should be adjusted to tackle these limitations.  Please see Lines 503 – 510.  In general, we observed temporal clustering (seasonality) of infections following the rainy season (Figure S12). However, we have limited information about the severity of symptoms.  Please see Lines 174 – 175 and Figure S12. |
| ED-22 | L464 “six-fold” is based on the S-Ra2 (which seems to be a slight overestimate) or HAI (which seems to be comparison of non-comparable values)? | Our comparison was based on HAI. Since HAI integrates AUC and S-Ra2, we considered avoiding making direct comparisons of HAI and S-Ra2 from the week-village quantitative model. We have deleted the potentially misleading statement.  Please see Line 542. |
| ED-23 | L521 “Supplemental to the R package…” seems to be incomplete thought. | We have edited this sentence from “Supplemental to the R package used for the model.”  To:  “Furthermore, it may also be affected by the R package used for the model.”  Please see Line 614 – 615. |
| ED-24 | Supplementary information – S3 Random forest, was the hyper-parameter tuning done in an automated fashion via ‘ranger’? | As to our best knowledge, “ranger” package does not have a specific built-in function for tuning hyperparameters. We created a grid for tuning the 4 hyperparameters (number of variables, number of trees, percentage of bootstrap sample, and node size). After the best parameters were found, the final model was generated. We edited the Text S6 for clarity.  Please see Text S6. |
| Reviewer #1 | | |
| R1-01 | Some sections of the Methods would benefit from further details and clarifications. Line 150. Why did you choose 2009-2013 for the model? Was more recent data not available? Please provide a little more detail on the rationale for the chosen time period. | Unfortunately, more recent data was not accessible during the study, limiting our analysis to this specific timeframe. We have added this limitation to the manuscript.  Please see lines 618 – 622. |
| R1-02 | Lines 237-245. The description of these temporal models is confusing. How do they differ from the previously described six temporal-spatial combination models?  Also, which ML algorithm(s) were used for these models? | Thank you for pointing out the unclarified methods description. The datasets used to implement the six spatio-temporal models were the combination of all spatial locations with all time points. For example, the week-village dataset integrated data from all 437 villages in 260 weeks (2009-2013) resulting in 113,620 observations. On the other hand, the temporal models were implemented with isolated data for each individual village (n = 437) for a total of 260 weeks. Therefore, each of the 437 models used only temporal data (weekly) from the particular village. We have improved the description in this section.  Please see Lines 269 – 279.  In the temporal models, we exclusively employed CIF due to its comparatively superior predictive accuracy, as demonstrated in the preceding step involving six spatiotemporal resolutions. Additionally, since the primary objective of this step was to discern variations in dengue predictability at each specific village, we deemed it unnecessary to construct 874 models (437 for quantitative and 437 for qualitative) using six algorithms. Developing such a multitude of models would be redundant, given that the overarching conclusion — "predictability among villages is different" — could be adequately inferred without the need for an extensive number of models.  Please also see our comment on ED-13. |
| R1-03 | Line 275. What was your rational for using a different train/test method for the hybrid model (80/20 split of 2009-2013 data) vs. the qualitative and quantitative models (2009-2012 train and 2013 test)? Comparison of model performance resulting from different test/train methodologies seems potentially problematic. | We apologize for the confusion generated by our statement. In the hybrid approach, we used the same splitting method as in the qualitative and quantitative models (2009 – 2012 for train and 2013 for testing). Data from 2009 – 2012 corresponds to 90,896 out of 113,620 observations (80%) while 2013 corresponds to 22,724 out of 113,620 observations (20%). Because of it was confusing, we edited the text about the splitting method for the hybrid approach for consistency in the manuscript.  Please see Line 315. |
| R1-04 | Line 277. The way you’ve appeared set up the hybrid model means that it could not be used in a predictive manner. The description seems to indicate that the quantitative models in the second step is only applied to locations where nonzero case counts occurred (not locations predicted to have cases from step 1). This seems like a big limitation to the future use of the proposed hybrid model. Fitting a quantitative model in all locations where the qualitative model indicated the presences of cases could overcome this limitation. | Thank you very much for pinpointing the confusing statement in the former Line 277. Our approach was designed to ensure the models are predictive. We fitted the quantitative model and predicted dengue incidence in all locations where the qualitative model indicated the presence of cases.  We have resolved this issue by rephrasing the sentence. Please see Lines 317 – 318. |
| R1-05 | Based on Line 360, this may actually have been what was done. Please clarify. | Thank you. We hope our response on R1-04 above helps to clarify this matter. |
| R1-06 | Line 290. Did you consider evaluating the hybrid models in a similar manner as other hurdle models? Absence locations can be treated as zeros so the full output from both steps can be evaluated as a quantitative model (e.g., calculate RMSE, R2-values). This could be an additional metric to compare to your proposed HAI metric. | Thank you for the suggestion. However, merging both predictions and calculating a single RMSE or R2 in the current hybrid approach setup is not feasible. The two steps utilize independent models with distinct parameter configurations, and combining predictions would mix data of different types. Additionally, the first step's predictions include false negatives, where actual values are significantly higher than zero. The Authors are cautious about estimating RMSE or R2 by comparing binary predictions (zero) with continuous actual values, as it may not be a fair approach. |
| R1-07 | Consider developing an additional figure which would visually present the model comparison results from the Tables in the main text. This would aid the reader in comparing accuracy across modeling techniques and spatio-temporal scales, a major takeaway of your manuscript. | Thank you for the suggestion. We have generated a bar plot figure demonstrating the accuracy values across all quantitative and spatiotemporal models presented in the Table 1 and 2.  Please see Fig. 2. |
| R1-08 | Conclusions are supported by the presented results. Contextualization of the proposed hybrid model in terms of currently used hurdle, mixture, and zero-inflated modeling frameworks would improve the discussion. | Thank you for the suggestion. We related our hybrid model to the existing zero-inflated and hurdle models in the abstract (please see Lines 47 – 48), Author summary (please see Lines 63 – 64), Introduction (please see lines 124 – 130 and 136 – 137), discussion (please see Lines 571 – 579), and conclusions (please see Line 633 – 634). |
| R1-09 | Line 172 (and others). Your indication of P-values could be misinterpreted as being truly zero. It would be more appropriate to indicate these values as something like <0.001. This would also apply to the Tables. | Thank you very much. We have edited the signals accordingly, replacing “=” with “<” for those p-values indicated as equal to 0.000.  Please see Lines 195 – 196, Lines 206 – 207, Table 3, and Table S4, |
| R1-10 | Line 208. You state “weeks” twice in this sentence. Likely you meant “month” for the seven time points. | Thank you. There was a mistyping in this sentence. The second ‘weeks” should be “months”. We have resolved the confusing statement.  Please see Lines 241. |
| R1-11 | Francisco et al. applied six machine learning algorithms to predict dengue cases in Metropolitan Manila in 2013. Their comparison of performance across spatio-temporal scales and between algorithms are highly valuable contributions. The authors also described a hybrid modeling approach to first predict the presence/absence of cases and then the number of dengue cases in “present” locations, finding an increase in accuracy with this method. While this framework may be new when applied to machine learning models, this is not a conceptually new framework; hurdle, mixture, and zero-inflated techniques are currently employed across a range of settings. Providing some discussion and comparison of the proposed hybrid model with such frameworks would frame the discussion. | Thank you for the valuable suggestion. Yes, hurdle model models and zero-inflated models can be comparable with our proposed hybrid approach. However, there is a key difference. While hurdle and zero-inflated models are designed to deal with zero-inflated data, they are limited to count data (Poisson distribution). Our hybrid approach can be applied in both Poisson and continuous data (Gaussian distribution). Furthermore, what sets this study apart is its pioneering effort to integrate machine learning within this framework of two step model.  We related our hybrid model to the existing zero-inflated and hurdle models in the abstract (please see Lines 47 – 48), Author summary (please see Lines 63 – 64), Introduction (please see lines 124 – 130 and 136 – 137), discussion (please see Lines 571 – 579), and conclusions (please see Line 633 - 634). |
| Reviewer #2 | | |
| None | | |
| Reviewer #3 | | |
| R3-01 | The authors present the methods clearly, the methods are well thought out and include a defensible and reasonable combination of models and tests for accuracy. The statistical analysis is robust. | Thank you for your positive feedback. |
| R3-02 | It might be nice to have slightly more description of the "quantitative" model, where all the zeros are taken out. | Thank you. To make the explanation much clearer, we have added a new figure to explain the conceptualization of the hybrid model.  Please see Fig. 1. |
| R3-03 | Does this allow for the quantitative model using previous dengue case counts as one potential predictor for future cases? | Appreciate your inquiry. Utilizing the target variable in a lagged fashion as a predictor is a feasible approach, commonly applied in autoregressive models like ARIMA. Nevertheless, to prevent the model from being excessively tailored to the dengue cases data (overfitting), the Authors opted to solely incorporate climatic and landscape features as predictors for dengue incidence. |
| R3-04 | The results are clearly presented and match the analysis as described. | Thank you. |
| R3-05 | Yes, the conclusions are clear and described well. The authors provide a creative and really useful hybrid approach that clearly improves predictions for dengue, particularly at smaller spatial/temporal scales. | Thank you |
| R3-06 | A few minor typos in references, and I don't think you need to describe the words "spatial" and "temporal" in the first paragraph (i.e. take out the parentheses) | We have fixed the typos in the references accordingly. |
| R3-07 | In general, this is a useful, creative approach to examining the spatial-temporal scales at which different models are more accurate and using a new hybrid approach that the authors developed to leverage the accuracy of the "qualitative" 0-1 models with the more useful case counts predictions of quantitative models that don't do well with a lot of zeros. Very cool!! | Thank you for your positive feedback! We are glad you found our approach to examining spatial-temporal scales and leveraging the accuracy of both qualitative and quantitative models interesting and useful. Your encouragement is greatly appreciated. |
