# Supplementary material for: Hybrid Machine Learning Approach to Zero-Inflated Data Improves Accuracy of Dengue Prediction
Source: PLoS Negl Trop Dis. 2024 Oct 21;18(10):e0012599. doi: 10.1371/journal.pntd.0012599 (PMC11527386; doi:10.1371/journal.pntd.0012599)
Supplement: Attachment — Submitted filename: PLOS NTDs_Response to the review v2.docx [file pntd.0012599.s015.docx]

Dear Editor,

Attached herewith is the revised version of our manuscript entitled: **Hybrid Machine Learning Approach to Zero-Inflated Data Improves Accuracy of Dengue Prediction.** We modified the manuscript following the suggestions made by the Editors and the two Reviewers. We answered and explained point by point the issues, comments, and suggestions made by the Editor and Reviewers. The manuscript has been revised accordingly with track changes and highlights for your guidance. Since there are numerous comments, each comment was provided with codes. The line numbers indicated in the table below correspond to the line numbering in the manuscript file without track changes. Since the questions and comments from the Editor and Reviewers differed, we have created within the table specific sections for the Editor and Reviewers to facilitate locating the responses.

We are very thankful to the Editor and Reviewers because their valuable insights made our manuscript better. We are hoping that our revisions will merit the approval for publication in your prestigious journal.

Thank you!

| Code | Question | Answer |
| --- | --- | --- |
| Editors | | |
| ED-01 | Please consider moderating claims about the high degree of novelty or pioneering nature of this paper, as some seem exaggerated. Incremental progress has value, and we agree that this study is a useful advance. Modeling and prediction of dengue at various spatio-temporal scales with a wide range of model forms, including zero-inflated and machine learning models, have been published previously. It is somewhat misleading to claim that "this is the first instance in which dengue prediction accuracy has been compared across various spatiotemporal resolutions." Even in the unlikely event that this is true, collectively many other studies on dengue prediction have evaluated predictive performance across a very wide range of spatio-temporal resolutions. | Thank you for your feedback and the opportunity to clarify our claims regarding the novelty of our study. We recognize the extensive body of research on dengue modeling and prediction across various spatio-temporal scales and using diverse model forms. Our study focuses on providing a useful advance by focusing on the integration of binary and continuous outcome models, which we believe has the potential to offer more robust predictions. Therefore, in light of your feedback, we have moderated our claims about the pioneering nature of the study. We have revised the manuscript to better reflect our contributions. Here is the revised statement:  “This study introduces a hybrid approach that combines both binary and continuous outcome modeling to enhance predictive accuracy in dengue forecasting. This method builds upon and complements existing methodologies such as zero-inflated and hurdle models”. Please see lines 47, 448 – 450 and 664 – 665. |
| Reviewer #1 | | |
| R1-01 | Line 240. Using the whole timeseries in the cross-correlation analysis could have inadvertently “leaked” information from the training to the testing set, thus artificially improving the testing performance of the models. Why was the whole timeseries used and how did you check you had not introduced this bias? | Thank you for your insightful comment regarding the potential for "information leakage" in our cross-correlation analysis using the whole timeseries data from 2009 to 2013. We understand that using the entire dataset for the cross-correlation could raise concerns about inadvertently improving the model's performance on the test set. We would like to clarify our approach and the rationale behind it. The primary objective of our correlation analysis was to identify and understand the lagged effects of environmental factors on dengue incidence. To achieve this, we conducted a cross-correlation analysis on the entire dataset to explore the relationships between environmental variables and dengue cases over time. This analysis aimed to uncover the lags at which these factors most strongly correlate with dengue incidence, which is essential for constructing a model with meaningful predictive features. We employed a temporal cross-validation approach to assess the robustness of the model. This involved splitting the data into multiple folds in a way that preserves the temporal ordering of the data. By training on past data (2009-2012) and validating on future data (2013), we ensured that the model's predictive performance was evaluated on unseen data that was not used during the training phase. The cross-correlation analysis served only as an exploratory step to identify potential lags, and the actual predictive model did not have access to the 2013 data during training. By employing these methods, we ensured that the model's predictive capabilities were based on patterns and relationships learned from the training data, without directly incorporating information from the test set. Our approach aligns with the standard practices in time series analysis, where understanding lagged effects is crucial for forecasting. We believe that this methodology provides a robust and reliable framework for predicting dengue cases based on environmental factors. We have edited the manuscript and added lines 242 – 246 for clarification. |
| R1-02 | Line 384. The statement about model improvement with merged village datasets could have some nuance. While most models showed improvement, the performance of the RF qualitative model appears to have decreased from non-merged to merge village scales. The SVM and XGB models each had a metric show reduced performance with the merged village datasets. | Thank you for the important observation. We have edited this section to: “Merging small villages in the Week-village data to increase the variance in predictor values (i.e. precipitation) improved the predictive accuracy for quantitative models with all algorithms except for the qualitative models. For example, CIF improved from S-Ra² = 0.116 to 0.220 for the quantitative model and from AUC = 0.794 to 0.806 in the qualitative model with merged villages. Similar improvements were also observed with GAM and ANN (Table S8 and Table S9). However, with qualitative models, RF, SVM and XGB showed reduced accuracy with aggregated data”. Please see lines 418 – 424. |
| R1-03 | Line 395. Did any village-scale temporal models have an AUC < 0.5 (worse than random chance)? The binning of AUC for equal width low, moderate, and high groups means that this cannot be ascertained from the text or Figure 3. If all villages had an AUC > 0.5, I would suggest truncating the low category at 0.5 (or whatever minimum AUC was observed) for clarity to the reader on the lower end of model performance achieved across villages. | Thank you for the constructive comment and suggestion. We have decided to truncate the low category at 0.5, assuming any village with AUC at such threshold could not be predicted. Then, set medium AUC from 0.5 – 0.75 and high from 0.75 – 1.00. We have edited the manuscript accordingly, please see lines 393 – 396 and 426 – 428, and also edited the Fig. 3 and Figure S10. |
| R1-04 | Line 472. An epidemic of dengue (not just a pandemic) could also reduce the frequency of zero cases. | Thank you for the observation. We have edited the sentence to “In epidemiological data on infectious diseases, especially at fine spatial and temporal resolutions, the frequency of zero values is typically high, except during a pandemic or when the disease becomes epidemic”. Please see lines 503 – 505. |
| R1-05 | The Discussion would be enhanced with some discussion around the biological plausibility of identified environmental covariates and dengue incidence (Tables S4 and S5). The authors mention associations with village size and population size, but do not appear to discuss the environmental covariates. | Thank you for your suggestion. We have added a brief discussion on the biological plausibility of the identified environmental covariates and their association with dengue incidence:  “Our study utilized climatic and landscape data, which are well-established and effective predictors of dengue incidence. Climatic factors significantly impact the transmission of dengue by influencing the life cycle and behavior of mosquitoes. For instance, higher temperatures accelerate the mosquito life cycle and virus replication, reducing the extrinsic incubation period and increasing the risk of outbreaks. Similarly, environments with high relative humidity, especially above 60%, promote mosquito survival and activity, thereby increasing their contact rate with humans. Rainfall creates temporary water bodies ideal for mosquito breeding, but excessive rainfall can wash away larvae, reducing populations temporarily. Conversely, a lack of rainfall often results in water being stored in containers, which can become mosquito breeding grounds if not properly managed. In residential areas, high human density, along with water storage and discarded items, enhances mosquito-human contact and breeding opportunities. Furthermore, improper waste disposal and water storage in commercial and industrial areas can also lead to breeding sites, with the movement of people between these and residential areas facilitating the virus’s spread. Even though less directly linked, vegetated areas like parks and forests can still harbor mosquito populations, particularly if they contain water features that support mosquito breeding. Although our data incorporated significant predictive factors for dengue incidence, achieving high accuracy at fine spatial scales proved challenging due to other factors discussed below”. Please see lines 459 – 476. |
| R1-06 | L206-207 (and following): The presentation of P-values as p<0.000 here and throughout the manuscript could appear that P-values were less than zero, which is impossible. Presenting them as p<0.001 would prevent this confusion. I defer to the journal guidelines on number of significant figures to include for these highly significant instances. | Thank you. We have edited for all p<0.000 to p<0.001 throughout the manuscript, including supplementary materials. Please see lines 199 – 201, 210 – 212, Table 3 and Table S4. |
| R1-07 | Thank you for your edits and additions to clarify your manuscript. It is much improved. The clarification throughout that zero-inflated and hurdle models cannot handle continuous data is a valuable addition to the manuscript to provide clear justification and motivation for your ML method. Additionally, Fig S3 is a very useful diagram to outline the data acquisition, pre-processing, and modeling flow. Thank you for including this. | Thank you for your positive feedback and constructive suggestions. We appreciate your acknowledgment of the improved clarity of our manuscript and we are also glad that Fig S3 effectively outlines the data acquisition and modeling process. |
| R1-08 | I have a few minor comments remaining (above sections and one below here). Line 128. This explanation is true for a hurdle model, but not for zero-inflated model. In a hurdle model, all zero observations are produced by a single piece of the model (binary piece). In a zero-inflate model, zeros can arise from the binary piece of the model as well as from the count piece of the model. The structure of the hybrid model appears to align with that of a hurdle model where the quantitative piece never produces any zeros. A zero-inflated framework for the hybrid model would not necessarily be incorrect; zeros produced from the quantitative piece would relate to dengue cases being present, but not detected through passive surveillance. Clarification around hurdle vs. zero-inflated models in regards to the authors intent of their model would be appreciated. | Thank you for highlighting the distinction between zero-inflated and hurdle models. Our original statement was simplified to emphasize that there are established models, such as zero-inflated and hurdle models, designed to handle zero-inflated data. We are sorry for the potentially misleading statement. We have revised the manuscript to clarify this distinction.  “The hurdle model first employs a binary model to predict zero or positive responses and second, a truncated model for positive data. All zeros are generated from the binary model while the non-zero values by the truncated component. In contrast, zero-inflated models allow zeros to be produced by both the binary and count components”. Please see lines 127 – 130. |
| Reviewer #2 | | |
| R2-01 | My primary remaining question with regards to the approach used here deals with one of the arguments that the authors present as a primary reason for using their two-stage ML approach instead of a more "traditional" (i.e. parametric) hurdle model. Namely, in L125 the authors argue that zero-inflated and hurdle models are designed for count data, but not continuous data as used here. What is unclear is why the authors have chosen to model this data as incidence rate, since it is count data initially. I find the argument about continuous data to be superfluous unless the value of the incidence rate is established first. Without this argument the two-part ML approach and the authors performance metrics are still valuable and compelling. | Thank you for your insightful comment regarding our approach and the choice to model the data as incidence rates instead of traditional count data. In our analysis, we chose to model dengue data as incidence rates to account for variability in the population size across different geographic areas. By doing so, we aimed to ensure that the predicted values would reflect the relative risk of dengue in a given area rather than just the absolute number of cases, which can be influenced by population size. This approach allows for a more nuanced understanding of the data, facilitating comparisons between areas with different population sizes. We have added such explanation in the manuscript. Please see lines 177 – 182. |
| R2-02 | However, additional attention is brought to the incidence rate in L178 where the incidence rate is transformed with the transformation that is common for count data (log + 1), despite the fact that incidence rate will always be less than 1. I would like additional explanation or assurance with data exploration figures that this is not problematic. | The log +1 transformation was applied to stabilize the variance and normalize the distribution of the incidence rate data. This transformation was commonly used in previous studies to address the skewness and over-dispersion often present in data. While incidence rates are different from raw count data, the transformation helps to manage the variability and improve model performance. In our analysis, not all dengue incidence rates remained below 1; several values rose above 1 following the log +1 transformation in the week-village dataset. Please see the edited lines 182 – 184 to explain why the log transformation was used and Figure S11A and B which were already included in the previous submission that shows that the range of the log-transformed dengue incidence surpassed 1. |
| R2-03 | Finally, in line 520 the authors talk about quantitative models predicting count variables, which is sensible for disease data but not strictly in keeping with the focus on the incidence rate here. I would like the authors to better explain the reason incidence rate is preferable to the raw counts and point out that their analysis would work on either counts or incidence rate. It seems to me that their "hybrid model" could equally well be called a machine-learning hurdle model or similar. A hurdle model can be accurately seen as any model with two parts that do the things their models do here, although they have in the past been primarily two different generalized linear models. For example: <https://gbr01.safelinks.protection.outlook.com/?url=https%3A%2F%2Fhal.science%2Fhal-03739838%2F&data=05%7C02%7C%7Ccf7e191b9b7a4b871a8f08dcb29aa94b%7C84df9e7fe9f640afb435aaaaaaaaaaaa%7C1%7C0%7C638581624547485086%7CUnknown%7CTWFpbGZsb3d8eyJWIjoiMC4wLjAwMDAiLCJQIjoiV2luMzIiLCJBTiI6Ik1haWwiLCJXVCI6Mn0%3D%7C0%7C%7C%7C&sdata=Tl8Xte0GQjFOkOugu0GS2wREdtdHYn%2F1gNI0muUcbgU%3D&reserved=0> or <https://gbr01.safelinks.protection.outlook.com/?url=https%3A%2F%2Flink.springer.com%2Farticle%2F10.1007%2Fs00607-023-01224-3&data=05%7C02%7C%7Ccf7e191b9b7a4b871a8f08dcb29aa94b%7C84df9e7fe9f640afb435aaaaaaaaaaaa%7C1%7C0%7C638581624547496166%7CUnknown%7CTWFpbGZsb3d8eyJWIjoiMC4wLjAwMDAiLCJQIjoiV2luMzIiLCJBTiI6Ik1haWwiLCJXVCI6Mn0%3D%7C0%7C%7C%7C&sdata=bxqfap36%2Fn84SVsoeDUHi8DVy8l7IBl5sbJXeSJmnqs%3D&reserved=0> | Thank you for your careful revision. The word “count” in line 520 was a mistake. In the context of this manuscript, we wanted to say “continuous”. We have changed the word. Please see line 552.  We have explained the reason for using dengue incidence above. Please see R2-01  We have added the following in the manuscript: “Furthermore, given that machine learning algorithms can handle a variety of distributions, including Negative Binomial, Poisson, and Gaussian, we believe that the second step of our hybrid approach could be applied to count data. However, it’s important to note that count data was not used in this study. Future research might explore implementing the hybrid approach with count data and compare the outcomes with traditional hurdle models”. Please see lines 619 – 624. |
| R2-04 | L238 I assume this should be the highest (absolute) Pearson correlation coefficient (i.e. largest positive OR negative correlation)? | Thank you for the observation. Yes, this is correct. We have edited the sentence to: “We identified the best-lag time based on the highest absolute value of Pearson correlation coefficient and whether the p-value is ≤0.05”. Please see lines 247 – 248. |
| R2-05 | Reviewer #2: L348 Unclear that "this resolution" refers to the week-region model, could be misinterpreted. | Thank you for the observation. In this sentence, we refer to the week-region, not week-village as wrongly stated. We have fixed the sentence to:  “Although RF exhibited a lower capacity to elucidate the variance in dengue within the week-region model, it attained the lowest error at this resolution (RMSE = 0.019)”. Please see lines 358 – 360. |
| R2-06 | Reviewer #2: I thank the authors for their improvements to the manuscript. Various aspects of the methods, results, and discussion were well clarified and their responses to previous reviews were careful. | We sincerely thank you for your positive feedback and for acknowledging the improvements made to our manuscript. Your careful and constructive reviews have been vital in enhancing the clarity and quality of our work. |
| R2-07 | I had requested that the authors include the code used. The new version includes an example version of the code, but I don't think this matches the intention of the journal (<https://gbr01.safelinks.protection.outlook.com/?url=https%3A%2F%2Fjournals.plos.org%2Fplosntds%2Fs%2Fmaterials-software-and-code-sharing&data=05%7C02%7C%7Ccf7e191b9b7a4b871a8f08dcb29aa94b%7C84df9e7fe9f640afb435aaaaaaaaaaaa%7C1%7C0%7C638581624547503180%7CUnknown%7CTWFpbGZsb3d8eyJWIjoiMC4wLjAwMDAiLCJQIjoiV2luMzIiLCJBTiI6Ik1haWwiLCJXVCI6Mn0%3D%7C0%7C%7C%7C&sdata=e%2BY1ysIn4GH1z9xEzfCK08kPujc83Q%2FgBdTKDQWu9XQ%3D&reserved=0>) that the work be reproducible, in that it only includes one algorithm and does not include the full procedure for tuning for that algorithm. The solution is to include the actual analysis code with the data in the Dryad repository. | Thank you for your comment. We included the full code directly in the supplementary material. Please see the updated S7 Code. |
| Reviewer #3 | | |
| None | | |
